# Polygenic scores for estimated glomerular filtration rate in a population of general adults and elderly – comparative results from the KORA and AugUR study

**DOI:** 10.1186/s12863-023-01130-9

**Published:** 2023-05-25

**Authors:** Janina M. Herold, Jana Nano, Mathias Gorski, Thomas W. Winkler, Kira J. Stanzick, Martina E. Zimmermann, Caroline Brandl, Annette Peters, Wolfgang Koenig, Ralph Burkhardt, André Gessner, Iris M. Heid, Christian Gieger, Klaus J. Stark

**Affiliations:** 1 Department of Genetic Epidemiology, University of Regensburg, Franz-Josef-Strauß-Allee 11, 93053 Regensburg, Germany; 2 Institute of Epidemiology, Helmholtz Zentrum München-German Research Center for Environmental Health, Neuherberg, Germany; 3 Department of Epidemiology, Institute for Medical Information Processing, Biometry, and Epidemiology, Ludwig-Maximilians-Universität, Munich, Germany; 4 Department of Ophthalmology, University Hospital Regensburg, Regensburg, Germany; 5 Deutsches Herzzentrum München, Technische Universität München, Munich, Germany; 6 Institute of Clinical Chemistry and Laboratory Medicine, University Hospital Regensburg, Regensburg, Germany; 7 Institute of Clinical Microbiology and Hygiene, University Hospital Regensburg, Regensburg, Germany; 8 Institute of Genetic Epidemiology, Helmholtz Zentrum München, Neuherberg, Germany; 9 Research Unit Molecular Epidemiology, Institute of Epidemiology, Helmholtz Zentrum München-German Research Center for Environmental Health, Neuherberg, Germany

**Keywords:** Polygenic scores, PGS, General adults, Elderly, Kidney function biomarkers, eGFR_crea_, eGFR_cys_

## Abstract

**Background:**

Polygenic scores (PGSs) combining genetic variants found to be associated with creatinine-based estimated glomerular filtration rate (eGFR_crea_) have been applied in various study populations with different age ranges. This has shown that PGS explain less eGFR_crea_ variance in the elderly. Our aim was to understand how differences in eGFR variance and the percentage explained by PGS varies between population of general adults and elderly.

**Results:**

We derived a PGS for cystatin-based eGFR (eGFR_cys_) from published genome-wide association studies. We used the 634 variants known for eGFR_crea_ and the 204 variants identified for eGFR_cys_ to calculate the PGS in two comparable studies capturing a general adult and an elderly population, KORA S4 (*n* = 2,900; age 24–69 years) and AugUR (*n* = 2,272, age ≥ 70 years). To identify potential factors determining age-dependent differences on the PGS-explained variance, we evaluated the PGS variance, the eGFR variance, and the beta estimates of PGS association on eGFR. Specifically, we compared frequencies of eGFR-lowering alleles between general adult and elderly individuals and analyzed the influence of comorbidities and medication intake. The PGS for eGFR_crea_ explained almost twice as much (R^2^ = 9.6%) of age-/sex adjusted eGFR variance in the general adults compared to the elderly (4.6%). This difference was less pronounced for the PGS for eGFR_cys_ (4.7% or 3.6%, respectively). The beta-estimate of the PGS on eGFR_crea_ was higher in the general adults compared to the elderly, but similar for the PGS on eGFR_cys_. The eGFR variance in the elderly was reduced by accounting for comorbidities and medication intake, but this did not explain the difference in R^2^-values*.* Allele frequencies between general adult and elderly individuals showed no significant differences except for one variant near *APOE* (rs429358). We found no enrichment of eGFR-protective alleles in the elderly compared to general adults.

**Conclusions:**

We concluded that the difference in explained variance by PGS was due to the higher age- and sex-adjusted eGFR variance in the elderly and, for eGFR_crea_, also by a lower PGS association beta-estimate. Our results provide little evidence for survival or selection bias.

**Supplementary Information:**

The online version contains supplementary material available at 10.1186/s12863-023-01130-9.

## Background

Polygenic scores (PGSs) have been widely applied as parameters for the individual genetic predisposition for complex traits and diseases [1]. A PGS is defined as the sum of alleles associated with a certain disease or a disease-related biomarker, weighted by the effect size of each variant derived from genome-wide association studies (GWAS) [2]. PGSs have shown promising results to identify individuals at high genetic risk for complex diseases at a comparable level as carriers of monogenic mutations [3]. A typical complex disease is chronic kidney disease (CKD), with multiple genetic as well as non-genetic risk factors. Diabetes and hypertension are known risk factors for renal failure and kidney disease progression [4, 5]. CKD is one of the most common diseases worldwide with a prevalence of > 10% and high morbidity and mortality [6]. Kidney function is typically assessed by glomerular filtration rate estimated by serum creatinine (eGFR_crea_) or cystatin C (eGFR_cys_) [7, 8]. A recent large GWAS in 1,201,909 individuals has identified 424 genetic loci including 634 independent genetic variants associated with eGFR_crea_ (*P* < 5 × 10^–8^) [9]. GWAS summary statistics for eGFR_cys_ based on 460,826 individuals has been made available, but an independent set of genome-wide significant variants for eGFR_cys_ to build a PGS has not yet been generated. These GWAS on eGFR_crea_ and eGFR_cys_ included primarily individuals at the general adults’ age: the largest contributing study was UK Biobank (age range = 40–69 years).

The PGS based on the 634 variants had been applied to various studies of different age ranges: the proportion of the eGFR variance adjusted for age and sex that was explained by the PGS varied substantially: 9.3%, 5.8%, or 4.2%, in UK Biobank (*n* = 436,581, age range = 40–69 years), the Norwegian HUNT study (*n* = 26,254, age range = 20–99 years), or the German AugUR study (*n* = 1,105, age range = 70–95 years), respectively [9]. The reasons for the observed smaller genetically explained variance in elderly individuals remained elusive. The explained variance expressed as R^2^ is commonly used to assess the informative value of a PGS. It depends on the phenotype variance of the respective study, the size of the PGS association with the phenotype, and the variance of the PGS. The phenotype variance can vary across study populations due to differences in factors that are associated with eGFR like age, comorbidities, and medication intake. The size of the PGS association with eGFR may differ between general adults and elderly, when the effect sizes of some genetic variants in the PGS differ by age. The extent to what age modifies genetic effects on eGFR is unknown.

The PGS variance might differ between elderly and general adults when the elderly’s allele frequencies of the included genetic variants may be subject to a potential survival or selection bias [10, 11]: morbidity- or mortality-protective alleles might be enriched in the elderly. Since cystatin C has been discussed in conjunction with successful aging and longevity [12], comparing allele frequencies between general adults and elderly might be particularly relevant for a PGS for eGFR_cys_.

Hence, our aim was to investigate the amount of eGFR variance that can be explained by PGS in elderly compared to general adults. For this, we analyzed two kidney function biomarkers, eGFR_crea_ and eGFR_cys_, which both estimate glomerular filtration rate, in individuals from two comparable studies capturing different age ranges: AugUR (Altersbezogene Untersuchungen zur Gesundheit der Universität Regensburg*,* age ≥ 70 years) [13] and KORA S4 (Kooperative Gesundheitsforschung in der Region Augsburg, age 24–69 years) [14]. Using GWAS summary statistics from Stanzick et al. [9], we derived independent genome-wide significant variants associated with eGFR_crea_ or eGFR_cys_ and the respective allelic effect sizes. In the AugUR and KORA S4 individuals, which were independent studies from the variant identifying GWAS, we computed the PGS based on these variants and compared the PGS association with eGFR between the studies.

## Results

### Participant characteristics

When restricting the AugUR and KORA S4 study data to individuals with available eGFR_crea_ or eGFR_cys_ assessment and genetic information, the analyzed samples yielded 2,900 KORA S4 participants and 2,272 AugUR participants. We compared the participant characteristics and found the prevalence of diseases and medication intake to be higher in the study of elderly individuals and the mean eGFR to be lower (Table 1).

**Table 1:** Participant characteristics in AugUR (*n* = 2,272) and KORA S4 (*n* = 2,900). Shown are the characteristics of the AugUR study where all individuals are > 70 years old and the KORA S4 study restricted to participants < 70 years, in order to have a fully distinct age range in the two otherwise comparable studies. Values are given as mean with standard deviation or as percentage and number; range of phenotype trait is given for all continuous traits

	AugUR		KORA S4	
Characteristic	Mean ± SD	Min, Max	Mean ± SD	Min, Max
Age [years]	78.4 ± 5.0	70, 95	46.2 ± 12.6	24, 69
Women	51.5% (*n* = 1,170)	-	52.8% (*n* = 1,532)	-
BMI [kg/m^2^]^a^	27.7 ± 4.5	15.66, 52.99	26.8 ± 4.7	15.8, 55.1
Education [years]^b^	12.3 ± 3.4	6, 23	11.79 ± 2.64	8, 17
Never smoking	55.5% (*n* = 1,258)	-	41.1% (*n* = 1,193)	
Former smoking	39.2% (*n* = 888)	-	31.7% (*n* = 919)	
Current smoking	5.3% (*n* = 119)	-	27.2% (*n* = 788)	
CAD^c^	15.5% (*n* = 351)	-	1.7% (*n* = 49)	-
Diabetes^d^	20.9% (*n* = 474)	-	3.1% (*n* = 89)	-
Antidiabetics	16.3% (*n* = 371)	-	2.6% (*n* = 74)	
Hypertension^e^	72.7% (*n* = 1,651)	-	32.0% (*n* = 927)	-
Antihypertensives	67.5% (*n* = 1,534)	-	13.6% (*n* = 395)	-
Heart failure^f^	14.7% (*n* = 335)	-	2.2% (*n *= 64)	-
High-ceiling diuretics	12.8% (*n* = 290)	-	1.3% (*n *= 38)	
Creatinine (serum) [mg/dL]	0.9 ± 0.3	0.4, 5.2	0.8 ± 0.2	0.4, 2.2
eGFR_crea_^g^[mL/min/1.73m^2^]	67.7 ± 15.9	9.9, 106.7	95.3 ± 15.5	24.3, 133.9
Cystatin C (serum) [mg/L]	1.2 ± 0.3	0.7, 5.2	0.8 ± 0.2	0.14, 3.3
eGFR_cys_^h^ [ mL/min/1.73m^2^]	60.9 ± 16.8	8.5, 106.3	99.2 ± 18.1	15.4, 248.5
CKD^i^	29.6% *(n* = 673)	-	1.5% (*n* = 43)	-
PGS^j^ (eGFR_crea_, unweighted)	627.8 ± 15.2	579.7, 679.0	626.4 ± 14.8	571.9, 681.1
PGS^k^ (eGFR_cys_, unweighted)	204.6 ± 8.8	175.8, 229.1	204.5 ± 8.7	173.2, 235.2
PGS^j^ (eGFR_crea_, weighted)	614.7 ± 14.0	561.0, 661.5	613.2 ± 13.3	565.9, 665.9
PGS^k^ (eGFR_cys_, weighted)	212.2 ± 8.5	179.7, 238.7	212.2 ± 8.4	181.3, 245.5

^a^BMI = body-mass-index

^b^Education was derived from school years plus the years with job or university training

^c^Coronary-artery disease (CAD) was defined as self-reported history of myocardial infarction or percutaneous coronary intervention or coronary bypass surgery (AugUR) or as myocardial infarction treated as inpatient (KORA S4)

^d^Definition for diabetes was based on self-reported diabetes or intake of antidiabetics

^e^Hypertension was defined based on the measurements at the study center and/or intake of anti-hypertension medication (excluding high-ceiling diuretics)

^f^Heart failure (HF) was assessed by self-reported presence of the disease (AugUR) or self- reported treated heart failure (KORA S4)

^g^eGFR_crea_ = estimated glomerular filtration rate using creatinine values (CKD EPI 2009)

^h^eGFR_cys_ = estimated glomerular filtration rate using cystatin C (CKD EPI 2012)

^i^Chronic kidney disease (CKD) was determined as eGFR_crea_ < 60 mL/min/ 1.73 m^2^

^j^PGS (eGFR_crea_, unweighted/ weighted) = polygenic score including 634 independent, genome-wide significant variants associated with eGFR_crea_; for KORA-S4 genotypes only 633 variants were available for PGS calculation. rs34188292 (MAF = 0.265, effect = -0.0019) was excluded

^k^PGS (eGFR_cys_, unweighted/ weighted) = weighted/ unweighted polygenic score including 204 independent, genome-wide significant variants associated with eGFR_cys_

### PGS distribution in elderly and general adults

Based on published GWAS summary statistics for eGFR_crea_ and eGFR_cys_ adjusted for age and sex [9], we selected the known 634 independent genome-wide significant variants for eGFR_crea_ and identified 205 independent genome-wide significant variants for eGFR_cys_ (634/633 and 204/204 available in AugUR/KORA S4, Supplementary Table 1). To compute the PGS for eGFR_crea_ and for eGFR_cys_ (PGS_eGFRcrea, PGS_eGFRcys) in each KORA S4 and AugUR participant, we counted the eGFR-lowering alleles, weighted each count by the allelic effect size determined in GWAS, and divided this by the sum of the weights. By this, one unit of the weighted PGS refers to one allele of average effect on eGFR.

**Fig. 1:**
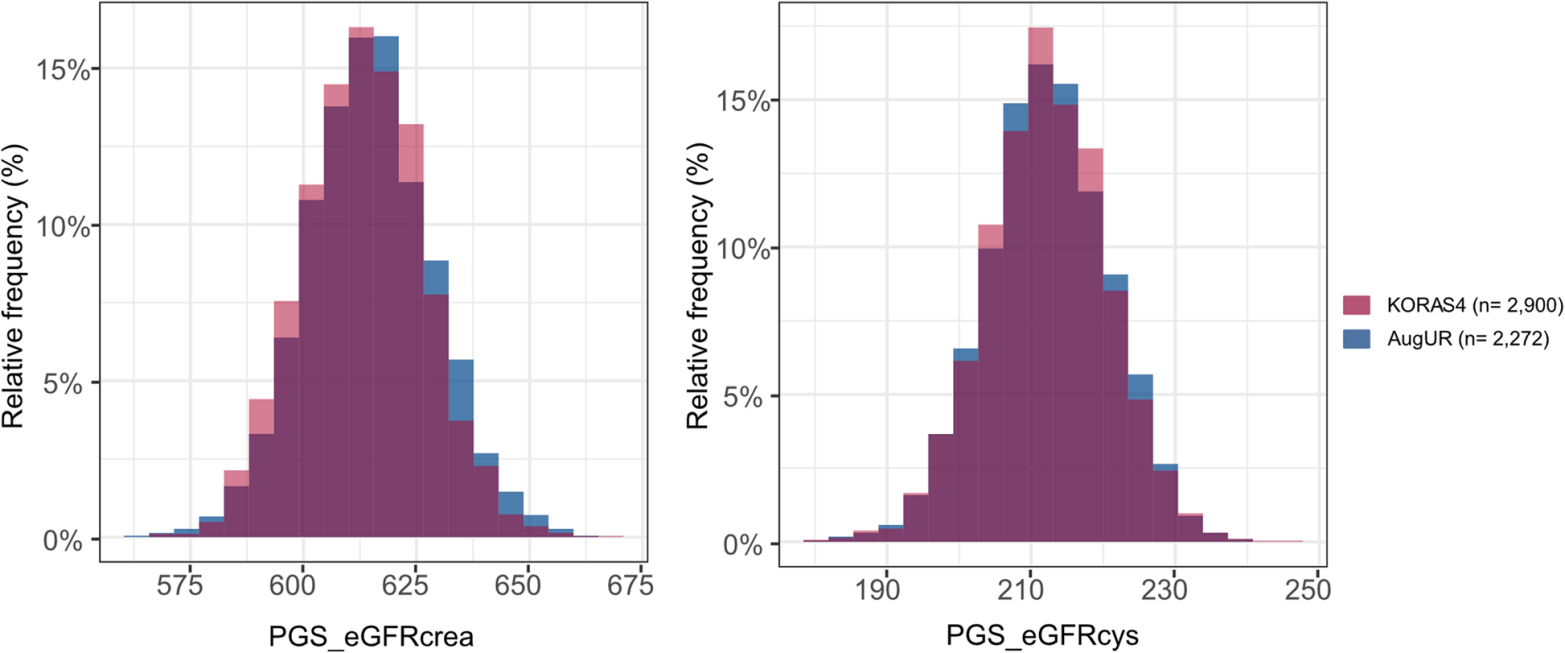
The distribution of PGS for eGFR in general adults and elderly. In each of the general adults (KORA S4, *n* = 2,900, magenta) and the elderly (AugUR, *n* = 2,272, blue), we show the distribution of the weighted PGS for eGFR_crea_ (**A**) or eGFR_cys_ (**B**). Overlapping intervals are coloured in dark purple. The y-axis shows the relative frequencies and the x-axis PGS_eGFRcrea or PGS_eGFRcys, respectively

We compared the PGS_eGFRcrea and PGS_eGFRcys distribution between the two studies (KORA S4 versus AugUR, *n* = 2,900 and 2,272). We found a similar distribution in the general adults compared to the elderly (Fig. 1) and a similar variance of the PGS (PGS_eGFRcrea: 14.0^2^ versus 13.3^2^, PGS_eGFRcys: 8.5^2^ versus 8.4^2^ in KORA S4 or AugUR, respectively, Table 1). The PGS was associated with eGFR by PGS categories in both studies, as expected (Supplementary Fig. 1).

### PGS-explained variance for eGFR adjusting for age and sex

We analyzed the PGS association with age- and sex-adjusted residuals of eGFR_crea_ and eGFR_cys_ by linear regression in each study: in the general adults, 9.6% of the age- and sex-adjusted eGFR_crea_ variance was explained by the PGS, and thus almost twice as high than the explained variance in the elderly (4.6%). This was similar for eGFR_cys_, but the difference was less pronounced (R^2^ = 4.7% versus 3.6%). The beta-coefficient for the PGS association was slightly higher in general adults compared to elderly for eGFR_crea_ (-0.29 [-0.33, -0.26] and -0.23 [-0.28, -0.19] mL/min/1.73m^2^ per one unit increase in PGS, respectively) and similar for eGFR_cys_ (-0.35 [-0.41, -0.29] versus -0.35 [-0.42, -0.27] mL/min/1.73m^2^, respectively). These and all further analyses were adjusted for ten principal components to account for potential study-specific sub-populations. Residual plots showed no evidence of non-linearity (Supplementary Fig. 2). The age-/sex-adjusted eGFR variance was smaller in general adults than in elderly (eGFR_crea_: 157.65 versus 228.93; eGFR_cys_: 186.38 versus 234.68 respectively). In general adults, the higher absolute genetic effect (beta of the PGS) on eGFR_crea_ and the smaller age-/sex-adjusted eGFR_crea_ variance could explain the larger R^2^ of the PGS for eGFR_crea_ compared to elderly. The larger R^2^ for eGFR_cys_ in general adults could be explained by the smaller age-/sex-adjusted eGFR_cys_ variance in general adults compared to elderly.

### Influence of age, sex, comorbidities, and medication intake on eGFR variance

**Fig. 2:**
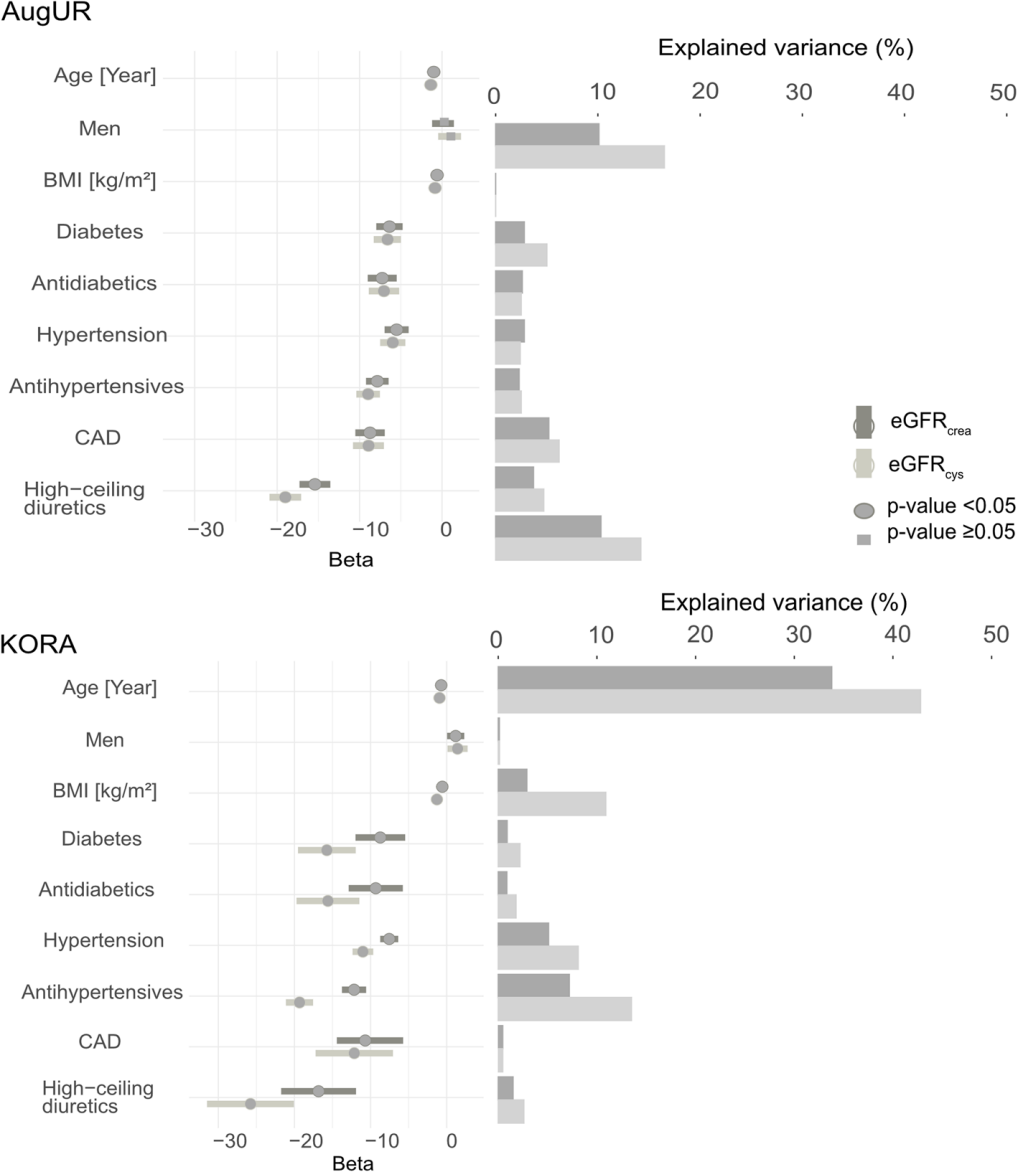
Influence of covariables on eGFR in the elderly and the general adults. Univariable linear regression was performed to estimate the association of each covariable (rows) with eGFR_crea_ and eGFR_cys_ in AugUR (**A**, *n* = 2,272) and KORA S4 (**B**, *n* = 2,900). Shown are beta-estimates and 95% CIs (left). Bar plots show the proportion of explained eGFR variance (R^2^) by each covariable (right)

We analyzed whether the observed differences in the PGS-explained variance between elderly and general adults were also due to differential frequencies of comorbidities or medication intake. We thus quantified the impact of comorbidities and respective medication intake on eGFR via univariable linear regression. Age showed a particularly high explained variance for eGFR in general adult (R^2^ = 34% for eGFR_crea_, 43% for eGFR_cys_, Fig. 2, Supplementary Table 2a, b). In the elderly, age (R^2^ = 10% for eGFR_crea_, 17% for eGFR_cys_) and high-ceiling diuretics intake (R^2^ = 10% for eGFR_crea_, 14% for eGFR_cys_) showed the highest explained variance. BMI explained more of the variance in eGFR_cys_ than eGFR_crea_ and more in the general adults compared to the elderly. A higher percentage of eGFR variance was explained by diabetes in the elderly compared to the general adults, in line with the higher diabetes prevalence in the elderly (20.9% versus 3.1%). Of note, our definitions of diabetes and antidiabetic intake are largely overlapping, as are the definitions of hypertension and anti-hypertensive intake. In summary, we observed a differential pattern of the impact of comorbidities and medication intake on eGFR between general adults and elderly.

### PGS-explained variance for eGFR after adjusting for comorbidities

We were interested in the PGS-explained variance of eGFR after adjusting for comorbidities. We compared the results from the previous model on age-/sex-adjusted eGFR (model 1) to the model further adjusting for BMI (model 2), then further for hypertension and diabetes (model 3) and then also adjusting for high-ceiling diuretics intake and CAD (model 4). The evaluated factors capture comorbidities related to eGFR: diabetes, hypertension, and CAD as well as increased BMI and heart failure, for which high-ceiling medication intake is a reasonable proxy. We found the residual eGFR variance decreasing by adjusting for these comorbidities, particularly in the elderly (Table 2). However, we found no notable impact by these adjustments on the beta-estimates of the PGS association with eGFR nor on the proportion of the variance explained by the PGS (Table 2).

**Table 2:** PGS association with eGFR in the elderly (AugUR) and general adults (KORA S4)

Model	Residual variance [ml/min/1.73 m^2^]^2^	Beta (PGS) [ml/min/1.73 m^2^] [95% CI]	*p*-value (PGS)	R^2^ (PGS) [%] [95% CI]
eGFR_crea_ AugUR				
Model 1	228.93	-0.23 [-0.28, -0.19]	4.80 × 10^–25^	4.6 [3.95, 5.25]
Model 2	218.72	-0.22 [-0.27, -0.18]	2.90 × 10^–24^	4.4 [3.80, 5.00]
Model 3	213.33	-0.23 [-0.27, -0.19]	1.07 × 10^–25^	4.7 [4.02, 5.37]
Model 4	198.70	-0.23 [-0.27, -0.19]	5.04 × 10^–29^	5.3 [4.47, 6.13]
eGFR_crea_ KORA S4				
Model 1	157.65	-0.29 [-0.33, -0.26]	5.44 × 10^–66^	9.6 [7.62, 11.58]
Model 2	157.18	-0.29 [-0.32, -0.26]	1.94 × 10^–65^	9.6 [7.62, 11.58]
Model 3	157.14	-0.29 [-0.32, -0.26]	1.89 × 10^–65^	9.6 [7.62, 11.58]
Model 4	156.37	-0.29 [-0.32, -0.26]	5.76 × 10^–66^	9.6 [7.62, 11.58]
eGFR_cys_ AugUR				
Model 1	234.68	-0.35 [-0.42, -0.27]	2.70 × 10^–20^	3.6 [3.19, 4.01]
Model 2	216.08	-0.31 [-0.39, -0.24]	3.60 × 10^–18^	3.2 [2.87, 3.53]
Model 3	211.93	-0.32 [-0.39, -0.25]	8.77 × 10^–19^	3.3 [2.95, 3.65]
Model 4	191.64	-0.31 [-0.37, -0.24]	2.34 × 10^–19^	3.5 [3.11, 3.89]
eGFR_cys_ KORA S4				
Model 1	186.38	-0.35 [-0.41, -0.29]	3.42 × 10^–32^	4.7 [4.10, 5.30]
Model 2	177.57	-0.35 [-0.40, -0.29]	1.24 × 10^–32^	4.7 [4.10, 5.30]
Model 3	177.29	-0.35 [-0.40, -0.29]	7.56 × 10^–33^	4.8 [4.18, 5.42]
Model 4	175.89	-0.34 [-0.40, -0.29]	1.14 × 10^–32^	4.7 [4.10, 5.30]

In AugUR (70–95 years, *n* = 2,272) and KORA S4 (20–69 years, *n* = 2,900), we derived the PGS association with eGFR_crea_ and eGFR_cys_ via linear regression,$Y_i\;=\;\beta_0+\;\beta_1\;PGS_i\;+\;\varepsilon_i,\;i\;=\;1,\dots,n$with $\varepsilon_i\sim\left(0,\sigma^2\right)$ independent and identically distributed. Y_i_ denotes the residuals of individual i adjusted for i) age and sex (model 1), ii) additionally for BMI (model 2), iii) additional for diabetes and hypertension (model 3), and iv) additionally for CAD and high-ceiling diuretics intake (model 4). All models were further adjusted for 10 principal components. Shown are the residual eGFR variance, the regression coefficients (beta) per one unit increase in the PGS with 95% confidence interval (CI) and *p*-values, and the R^2^ of the PGS. One unit in the PGS corresponds to one eGFR-lowering allele of average eGFR-effect

**Fig. 3:**
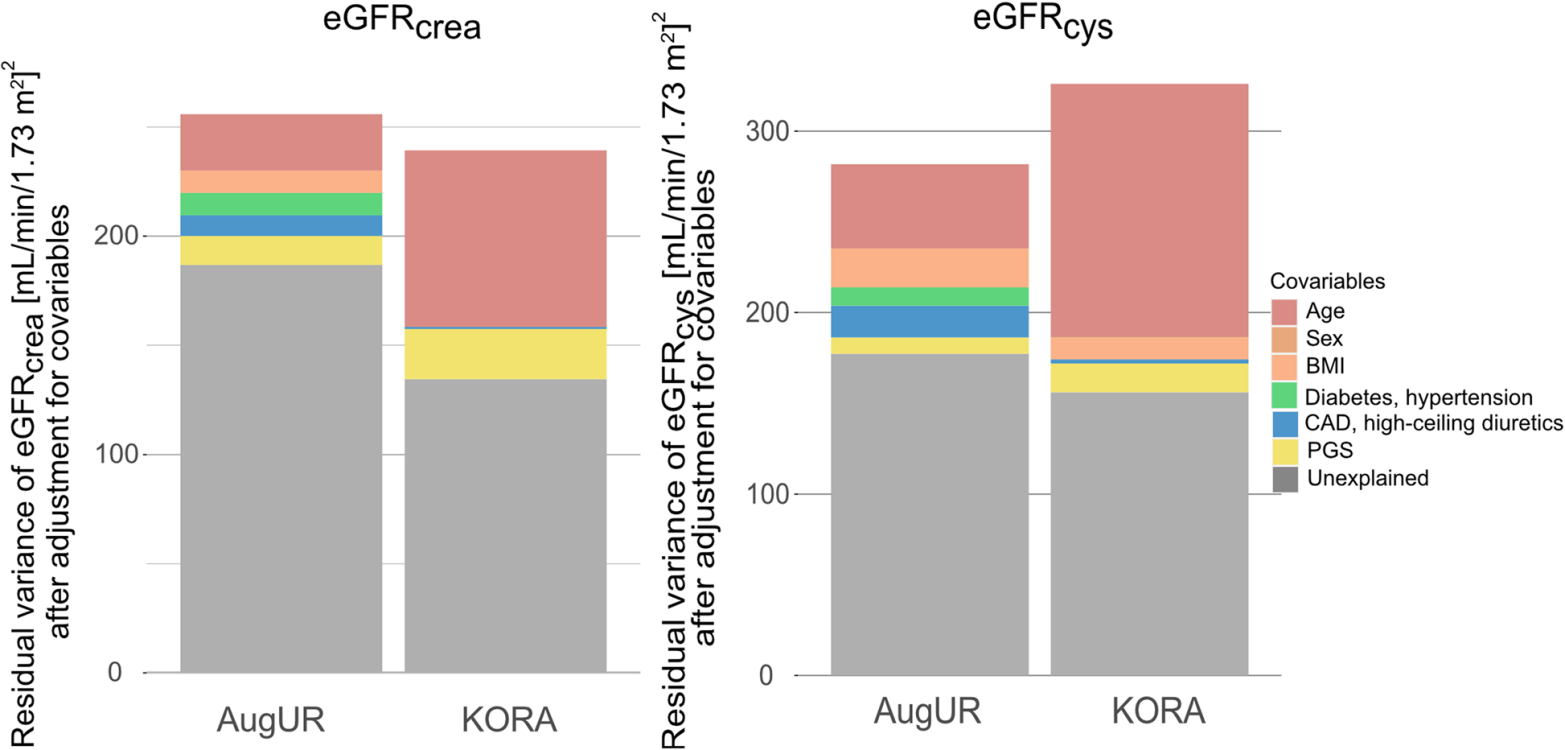
Residual eGFR variance after adjusting for age, sex, comorbidities and PGS in a stepwise fashion in elderly and general adults. We applied linear regression models on eGFR_crea_ and eGFR_cys_ increasing the number of covariables in a stepwise fashion: adjusting for age, sex, BMI, DM, hypertension, CAD, high-ceiling diuretics intake, and PGS. We show the residual variance of eGFR_crea_ (**A**) or eGFR_cys_ (**B**) in AugUR (*n* = 2,272) and KORA S4 (*n* = 2,900). The colored area is thus reflecting the proportion of the variance explained by the respective covariable adjusted for the covariables in the list above

Further evaluation of the impact of educational level or smoking revealed no impact on the PGS-explained eGFR variance (Supplementary Table 3). Altogether, the PGS explained more of the eGFR variance in the KORA S4 general adults than in the elderly AugUR individuals in all models. This was also observable when visualizing the residual eGFR_crea_ and eGFR_cys_ variance after adjusting for age, sex, and comorbidities in a stepwise fashion (Fig. 3).

When querying the 634 variants in the PGS_eGFRcrea and the 204 variants in the PGS_eGFRcys for their association in GWAS on diabetes, hypertension, or BMI at *p* < 5 × 10^–8^, we found up to 26 such variants (Supplementary Table 12). Some of these variants might exert their association on eGFR indirectly, mediated by a direct effect on diabetes, hypertension, or BMI. The PGS-association analyses adjusting for these comorbidities (model 2–4) showed similar beta-estimates for eGFR as model 1 (Table 2); lower or vanished beta-estimates would have indicated a mediating effect.

### Comparing the PGS variance and allele frequencies between general adults and elderly

We investigated whether differences in the PGS variance or differences in allele frequencies could explain the differential R^2^ values between the two studies. For the weighted PGS, we have shown above that PGS distributions were visually similar, PGS_eGFRcys variances were equal, and the PGS_eGFRcrea variance was slightly higher in the elderly. We observed the same pattern for the”unweighted” PGS (i.e. counting the number of eGFR-lowering alleles without weighting by GWAS-derived genetic effect estimates): (i) similar distributions upon visual inspection (Supplementary Fig. 3), (ii) equal PGS_eGFRcys variance (8.75^2^ versus 8.68^2^ in elderly or general adults, respectively) and slightly higher PGS_eGFRcrea variance in the elderly (15.17^2^ versus 14.82^2^); (iii) significantly different PGS_eGFRcrea distributions between elderly and general adults but no difference for PGS_eGFRcys (Mann–Whitney test *p* = 5.57 × 10^–4^ or 0.71, respectively). In summary, the PGS_eGFRcrea distributions – weighted or unweighted—were slightly different between general adults and elderly, but the PGS_eGFRcrea variance was smaller in the general adults and thus into the “wrong” direction to explain the larger R^2^ in general adults: R^2^ = beta^2^ * variance (PGS) / variance (outcome).

**Fig. 4:**
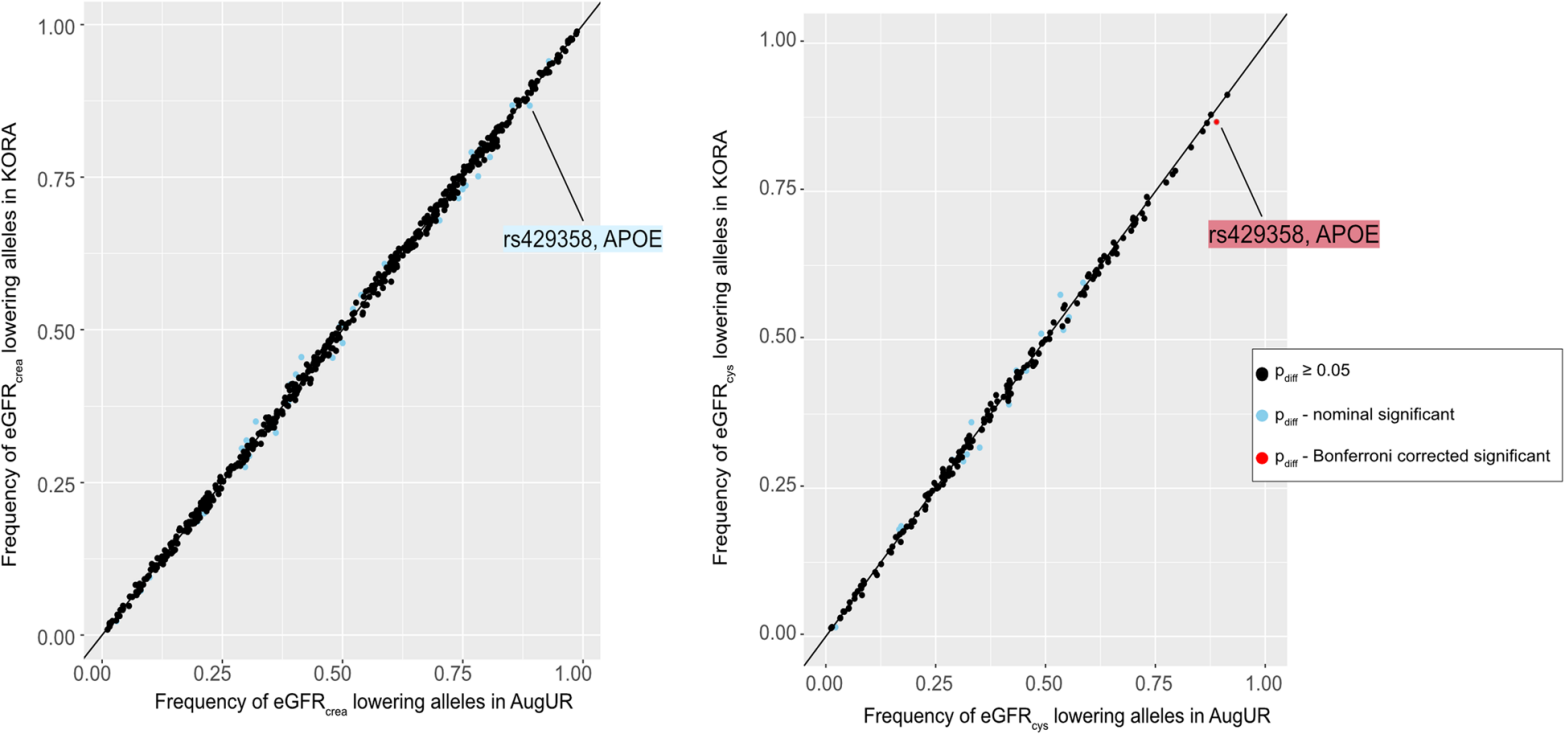
Frequencies of the eGFR-lowering alleles that are part of the PGS in the general adults compared to the elderly. Shown are the frequencies of the eGFR lowering alleles that are part of PGS_eGFRcrea (**A**) or PGS_eGFRcys (**B**) in the general adults (KORA, *n* = 2,272, y-axis) versus the elderly (AugUR, *n* = 2,900, x-axis). Here, we focused on the variants with high imputation quality (r.^2^ > 0.8; 534 in the PGS_eGFRcrea, 186 in the PGS_eGFRcys). We tested the allele frequencies for difference between the two studies (Chi-square test); color codes no (black), nominal (blue), or Bonferroni-corrected significance (red) of the difference (p_diff_ < 0.05/534 for variants in the PGS_eGFRcrea and p_diff_ < 0.05/186 for the variants in the PGS_eGFRcys)

Next, we compared the allele frequencies of each variant in the PGS for differences between general adults and elderly focusing on variants with high imputation quality (r^2^ > 0.8; 534 variants for PGS_eGFRcrea, 186 for PGS_eGFRcys) (Supplementary Tables 4 and 5). We found the allele frequencies to be very similar upon visual inspection (Fig. 4).

However, 37 variants in PGS_eGFRcrea and 17 variants in PGS_eGFRcys showed nominal significant difference between the two studies. In addition, one variant in PGS_eGFRcys showed Bonferroni corrected significant higher allele frequency in the elderly (rs429358 in *APOE* gene).

Furthermore, we observed an excess of small p_diff_-values compared to the expected p_diff_ (Supplementary Fig. 4). This was in line with an enrichment of variants with nominally significant allele frequency difference between the two studies (37 of 534 PGS_eGFRcrea variants and 17 of 186 PGS_eGFRcys variants with p_diff_ < 0.05; binomial enrichment for success probability ≥ 0.05 under the null: p_bin_ = 0.030 or 0.013, respectively). When looking at the effect direction of the variants showing allele frequency differences, we found no evidence for enrichment towards lower or higher eGFR-lowering allele frequencies in the elderly: among the variants with nominally significant allele frequency difference, 17 out of 37 in PGS_eGFRcrea and 8 out of 17 in PGS_eGFRcys had a lower frequency of the eGFR-lowering alleles in the elderly (binomial enrichment with success probability = 0.50 under the null: p_bin_ = 0.46 or 0.72, respectively).

We further explored the association of the genetic variant dosages with study membership or age in joint data pooling the two studies’ participants: we found an excess of small *p*-values of association with study membership or age, but not with age adjusting for study membership (Supplementary Fig. 5). Altogether, we observed an excess of genetic variants with subtle differences in allele frequencies and dosages between the two studies, but we did not find a systematic enrichment towards lower or higher frequencies of eGFR-lowering alleles in the elderly.

None of the tested variants in the PGS_eGFRcrea or PGS_eGFRcys, except one variant, showed a significant difference in allele frequency between general adults and elderly at a Bonferroni-corrected significance level (α = 0.05/534 = 9.36 × 10^–5^ or 0.05/186 = 2.69 × 10^–4^, respectively). Only the variant rs429358 in the PGS_eGFR_cys_, located in the *APOE* (apolipoprotein E) gene, showed a significantly higher frequency of the eGFR-lowering allele in the elderly compared to general adults (0.89 versus 0.87, respectively; p_diff_ = 1.53 × 10^–4^). This variant was also part of the PGS_eGFRcrea, but without Bonferroni-corrected significant difference due to the higher multiple testing burden when testing the 534 variants. In sensitivity analyses to understand the potential impact of the *APOE* variant on the PGS-explained age-/sex-adjusted eGFR variance, we generated a PGS without this variant. This yielded identical R^2^-values as observed for the original PGS (PGS_eGFRcrea R^2^ = 9.6% and 4.6%, PGS_eGFRcys R^2^ = 4.7% and 3.6% for general adults and elderly, respectively, Supplementary Table 6).

## Discussion

**Fig. 5:**
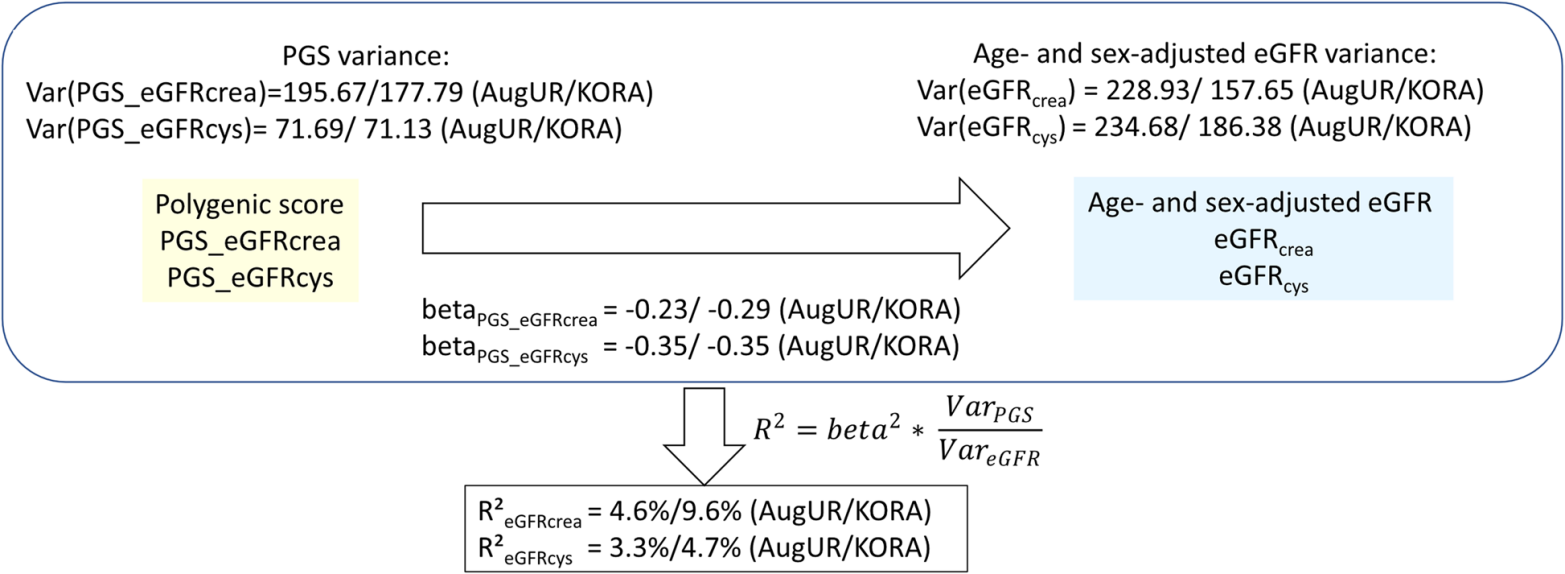
Scheme for calculation of explained eGFR variance by PGS. We computed the association of the PGS with eGFR residuals adjusted for age and sex in KORA S4 (*n* = 2,900, general adults) and AugUR (*n* = 2,272, elderly) via linear regression. Shown are the PGS-explained variance of eGFR_crea_ and eGFR_cys_ (R^2^) and the three components that drive the R^2^-values (relative genetic effect): PGS variance, beta-coefficient of the PGS-association with eGFR (absolute genetic effect), age-/sex adjusted eGFR variance

We built the PGS for eGFR_crea_ with 634 independent associated variants located at 424 broader genetic loci using the currently largest GWAS for eGFR_crea_ [9]. For the first time, we derived a PGS for eGFR_cys_ using the 204 variants with genome-wide significance from previously published GWAS summary statistics [9]. In our two studies, KORA representing the general adults aged 24 to 69 and AugUR with elderly participants above 70 years of age, we found the PGS-explained proportion of the age-/sex-adjusted eGFR_crea_ variance to be twice as high in general adults, 9.6%, compared to elderly, 4.6%. This difference was also observed for eGFR_cys_ – to a smaller extent. Our data suggested (i) that the difference in R^2^-values for eGFR_crea_ was due to the smaller age-/sex-adjusted eGFR variance and the higher beta-estimate of the PGS association on eGFR in the general adults compared to the elderly, and (ii) that the differences for eGFR_cys_ were due to the smaller age-/sex-adjusted eGFR variance, but the beta-estimates were the same (Fig. 5). Further adjustment for comorbidities reduced the residual eGFR variance particularly in the elderly, but exerted little impact on the PGS-explained eGFR variance and thus did not explain the difference in R^2^-values.

A limiting factor is the lack of elderly in GWAS generally [15]. This is also true for the GWAS for eGFR, which included primarily general adults that are more similar to the KORA than to AugUR participants. It is perceivable that the genetic variant effects are different in the elderly or that other genetic variants are responsible for kidney function differences in the aging kidney. The knowledge of the age-dependency of genetic effects on eGFR is limited and GWAS with genetic-by-age interaction analyses are warranted.

This might be particularly relevant for GFR assessed by creatinine comparing elderly with general adults. The creatinine metabolism is considered lower in elderly due to the decline of muscle mass in old age [16]. Unlike creatinine, cystatin C levels are known to be independent from age, sex, muscle mass, and ancestry and, therefore, considered as more eligible marker for GFR in elderly [17, 18]. The variant-identifying GWAS included predominantly general adults. It is known that some of the eGFR_crea_-associated variants are implicated in regulating creatinine metabolism rather than kidney function [19, 9]. These variants might have no or smaller effects on eGFR_crea_ in elderly due to lower muscle mass. For variants identified for eGFR_cys_, we would not expect such a difference. This would be in line with our findings: we observed a lower PGS beta-estimate for eGFR_crea_ in the elderly compared to general adults, beta = -0.23 or -0.29 mL/min/1.73 m^2^ per one unit increase in PGS, respectively, and a lack of such a difference for eGFR_cys_, both -0.35 mL/min/1.73 m^2^ per one unit increase in PGS. The difference in the PGS beta-estimate for eGFR_crea_ and the resulting difference in R^2^-values between elderly and general adults might thus be due to the underrepresentation of elderly in the identifying GWAS and a lack of gene-by-age interaction analyses.

Our models for eGFR as outcome with different adjustment revealed several interesting aspects: (i) Of all covariables, age explained the most of the eGFR_crea_ and eGFR_cys_ variance in the general adults. In the elderly, age and high ceiling diuretics intake had the strongest impact on the eGFR_crea_ or eGFR_cys_ variance. (ii) BMI showed a higher R^2^ for eGFR_cys_ than for eGFR_crea_. This might be explained by a the larger number of nucleated cells producing cystatin C resulting in increased cystatin C serum concentrations in individuals with higher BMI [20]. (iii) In the elderly, we found the residual eGFR_crea_ and eGFR_cys_ variance reduced by adjusting for diabetes, hypertension, CAD, BMI, and high-ceiling diuretics as a proxy for heart failure. However, these comorbidities did not explain the observed lower PGS-explained eGFR variance in the elderly compared to general adults.

An important aspect when performing PGS analyses in older individuals is a possible survival or selection bias leading to higher allele frequencies in the elderly for mortality- or morbidity-protective alleles [10, 11]. We found no enrichment of the eGFR-protective alleles in the elderly. We found no Bonferroni-corrected differences in allele frequencies between the two studies, except a significant difference for one variant located in the *APOE* gene (rs429358): the eGFR-lowering allele was more frequent in the elderly which argued against a survival effect. However, the same allele has been shown to be favorable for longevity [21] and protective for Alzheimer disease [22]. This was thus in line with a small survival bias and/or a selection towards the mentally healthy: AugUR participants had to come to the study center and answer all questions personally and were thus physically and mentally relatively healthy elderly. Considering that individuals with this allele tend to become older and older individuals have a lower eGFR, this is in line with this allele being associated with lower eGFR. The finding of a higher frequency of this *APOE* allele in elderly might be indicative of a survival or selection bias that is only indirectly related to impaired kidney function. In any case, this had no impact on the PGS-explained variance as shown by sensitivity analyses excluding this variant.

Some aspects on the variants’ independence and their directness or indirectness of effects on the phenotype should be noted: our PGS was generated based on genome-wide significant genetic variants identified by GWAS that was fully independent from our two studies here. The genetic variants included into the PGS were independent from each other based on conditional analyses using the GWAS data. We did not apply LDpred or related methods in our data to establish a PGS, since this would have required larger sample sizes and further independent data to test the PGS association [23, 24]. With regard to the directness or indirectness of variant effects, the *APOE* variant might depict an indirect genetic effect on eGFR via this variant’s association with longevity and thus higher age consistent with lower eGFR. Some other genetic variants in the PGS might capture further indirect effects: several variants were genome-wide significantly associated with diabetes [25], hypertension [26], or BMI [27]. These variants might have pleiotropic effects or exert an indirect effect on eGFR via these comorbidities as mediators. The similar beta-estimates of the PGS association with eGFR with and without adjusting for these comorbidities do not suggest such a mediating effect; however, an indirect effect might not have been completely removed given the typical underlying uncertainty in the diagnosis.

Some further limitations warrant mentioning: We observed some excess of variants in the PGS with subtle allele frequency differences between the two studies that might be beyond the differential age range. These differences might be random effects by separate genetic variant imputation or small selection bias for unknown reasons but are unlikely to explain the larger PGS-explained eGFR variance in general adults compared to elderly. Additionally, our PGS analyses were restricted to European individuals and may not be transferable to other ancestries. Another limitation might be the usage of eGFR equations that still include race-determining coefficients [7, 28], despite political defeasibility [29]. The reason to use these formulas here was the transferability of effect sizes estimated in the GWAS, which was also based on these CKD-Epi equations for eGFR [9]. Finally, the presence of diabetes has previously been shown to influence the effects of some SNPs on eGFR_crea_ [30]. Future analyses accounting for gene-environment interactions on eGFR will offer the opportunity to improve genetic effect size estimation and the variance explained by PGS in subgroups [2].

A major strength of our analyses is the high comparability between AugUR and KORA, since the two studies were designed jointly. The broad spectrum of drug classes and co-morbidities assessed in these two studies enabled the investigation of the influence of these covariables on the eGFR variance. To our knowledge, these analyses are unique in the investigation of PGS contrasting a population of general adults with an older population regarding differences in explained variance of eGFR.

## Conclusions

Our findings provide an idea for the reasons that lead to the observed differences in PGS-explained variance of eGFR between elderly and general adults. Our development of a PGS for eGFR_cys_ enabled to show that this difference was still apparent, but less pronounced for eGFR_cys_ than eGFR_crea_. We concluded that the difference was due to the higher age- and sex-adjusted eGFR variance in the elderly. This was independent of the utilized biomarker for GFR. For eGFR_crea_, there was another aspect: the lower R^2^-value was also explained by lower beta-estimates of the PGS association on eGFR_crea_ in the elderly. This might be due to limited representation of the elderly in the identifying GWAS, which impacts eGFR based on creatinine, but not cystatin. Our data provided little – if any – evidence for a genetically manifested survival or selection bias. Our analyses underscore the need of a critical view on R^2^-values and the corresponding components—eGFR variance, PGS variance, or PGS beta-estimates.

## Methods

### AugUR study sample

AugUR (*Altersbezogene Untersuchungen zur Gesundheit der Universität Regensburg*) is a research platform recruiting from the general mobile elderly population in and around Regensburg, a middle-sized city in the South of Germany with a study region capturing about 330,000 inhabitants of mostly Caucasian ethnicity [13]. The two AugUR baseline surveys conducted in 2013–2015 (AugUR1) and 2017–2019 (AugUR2) include 2,449 participants aged 70 to 95 years based on a random sample from the local registries of residence. Details on procedures and protocols of the AugUR study have been recently described [13, 31, 32]. A study sample of 2,272 AugUR participants with available eGFR values and genetic data was used for PGS analyses.

### KORA S4 study sample

The German KORA study (*Kooperative Gesundheitsforschung in der Region Augsburg*) is a population-based adult cohort study in the Region of Augsburg, Southern Germany, that was initiated in 1984 and comprises four surveys (S1-S4) with follow-up investigations on regular intervals [14]. In this study, we used data from the baseline S4 study visit, which included 4261 general adults, aged 25–74 years, recruited between 1999 and 2001 (S4). To allow comparison with AugUR study, the final sample was based on 2,900 participants (aged < 70 years) with available biomarker, covariables, and genotype data.

### Assessment of participant characteristics

Study design of AugUR was largely based on KORA S4 and thus assessment of characteristics was highly comparable. A detailed description of data collection in both studies is described elsewhere [13, 14, 33]. In brief, self-reported information on common diseases, medication intake and lifestyle factors were gathered via a standardized face-to-face interview. Medical exams by trained staff and laboratory measurements with standard procedures were conducted. Body-mass index (BMI) was computed based on measured weight divided by squared body height [kg/m^2^]. Hypertension was defined as actually measured systolic blood pressure of ≥ 140 mmHg, diastolic blood pressure of ≥ 90 mmHg according to general clinical standards [34] or corresponding medication intake indicating hypertensive blood pressure. Diabetes was assessed as self-reported diabetes or reported antidiabetic therapy intake. Variables for medication were gathered from medication charts, self-report or brought pill boxes.

### Assessment of kidney function in AugUR

Estimated glomerular filtration rate (eGFR [mL/min/1.73m^2^]) was derived from serum creatinine and cystatin C levels measured via enzymatic assay using the 2009 Chronic Kidney Disease Epidemiology Collaboration (CKD-EPI) creatinine Equation [35] and the cystatin equation from 2012 [28], respectively. Definition for chronic kidney disease (CKD) at eGFR_crea_ < 60 mL/min/1.73 m^2^ corresponds to current KDIGO (Kidney Disease: Improving Global Outcomes) guidelines [36].

### Assessment of kidney function in KORA S4

Estimated glomerular filtration rate (eGFR, [mL/min/1.73m^2^]) was derived from serum creatinine and cystatin using the 2009 Chronic Kidney Disease Epidemiology Collaboration (CKD-EPI) creatinine Equation [35] and the cystatin equation from 2012 [28], respectively. Creatinine concentrations were measured in milligram per deciliter via enzymatic assay and standardized to IDMS (Information Display Measurements Standard); cystatin C concentrations were measured in milligram per liter and based on nephelometry and were IFCC (International Federation of Clinical Chemistry) standardized. Validity of cystatin levels was verified and measured serum concentrations were categorized in four classes: within assay range (1), measured below detection limit (2), set below detection limit (3), set above detection limit (4). Values in categories 2–4 were excluded from PGS analyses.

### Genetic data in KORA S4 and AugUR

Genetic data from KORA S4 was described previously [33]. A detailed description of generating and processing genotype data in AugUR can be found in Supplementary Methods (Additional file 1). In both studies, the data is based on the imputation panel of the Haplotype reference consortium (HRC) and individuals in these analyses were restricted to unrelated and European individuals.

### PGS calculation

Based on published GWAS summary statistics on eGFR_crea_ adjusted for age and sex, we chose an established PGS based on independent genome-wide significant variants associated with eGFR_crea_ (*p*-value < 5 × 10^–8^, [9]). In line with that, we selected the set of independent genome-wide significant variants associated with eGFR_cys_ adjusted for age and sex. The independence of the genetic variants was obtained by selecting the wider, non-overlapping genetic loci, followed by conditional analyses using GCTA [37]. The weighted PGSs were generated by multiplying the allele dosage of each variant’s eGFR-lowering allele by its respective weight, then summing across all variants in the score, and dividing it by the sum of the weights using R version 3.6.3 [38]. Weights were derived by the genetic effect estimates from the GWAS for eGFR_crea_ and eGFR_cys_ [9]. For the unweighted PGSs, allele dosages were summed without considering the variant-specific effect sizes. An overview of the PGS analyses can be found in Supplementary Fig. 6 and further methodological details can be found in Supplementary Methods.

### Statistical analyses

For statistical analyses and plotting, we used R version 3.5.2 extended by packages such as *foreach* [39], *doParallel* [40], *data.table* [41], *stringr* [42], *scales* [43], *dplyr* [44], *Vcf Tools* [45], *gplots* [46] and *ggplot2* [47] as well as SPSS 28 (IBM SPSS Statistics for Windows, Version 28.0 Armonk, NY: IBM Corp.).

For the analysis of the association of the PGS on eGFR adjusted for age and sex, we derived the residuals of eGFR from linear regression on eGFR with age, sex, and ten genetic principal components as covariables. We then used these residuals as outcome in the following model: ${Y}_{i}={\beta }_{0}+{\beta }_{1}{PGS}_{i}+{\varepsilon }_{i}, i=1,\dots ,n$ with ${\varepsilon }_{i}$~(0,${\sigma }^{2}$) being independent and identically distributed and Y_i_ the residuals of eGFR for an individual i. This yielded the residual eGFR variance, the beta-estimate of the PGS on eGFR (per unit of the PGS) and the R^2^ that the PGS explains relative to the residual eGFR variance. We also derived the eGFR-residuals adjusting for further covariables and applied the same model as stated above to derive the PGS-association on these eGFR-residuals. The quality of models underlying linear regression is given by the R^2^-value, which results from variance decomposition. Details on R^2^ as parameter for explained variance are described in Supplementary Methods.

We tested the PGS distributions for differences between the two studies using the non-parameter Mann–Whitney U test. The Pearson’s chi-squared test was applied to test for differences in the frequencies of the eGFR-lowering alleles between AugUR and KORA S4. This analysis was based on best-guess genotypes derived from allele dosages. To enable higher accuracy, variants with an imputation quality < 0.8 were excluded for this analysis. We conducted two types of binomial enrichment tests: (i) We conducted a one-sided binomial test to infer whether the observed number of variants with observed nominally significant allele frequency differences was enriched compared to what would be expected under the null of "no differences in allele frequencies". Based on the observed number of successes (i.e., the number of variants with nominally significant allele frequency differences), the binomial test compares the null of "probability of success *p* <  = 0.05" with the alternative hypothesis of "probability of success *p* > 0.05" based on a Bernoulli experiment (in R: binom.test (k,n.p = 0.05,alternative = "greater")). (ii) We further conducted a two-sided binomial test to infer whether the number of variants with nominally significant allele frequency differences were enriched for a specific direction (i.e., whether the eGFR-lowering alleles were less or more common frequent in AugUR compared to KORA). We restricted this to the variants that showed a nominally significant difference between the two studies. Based on the observed number of successes (i.e., the number of variants where the eGFR-lowering alleles that were less frequent in AugUR), the binomial test compares the null hypothesis of “probability of success *p* = 0.5” with the alternative hypothesis of “probability of success *p* <  > 0.5” based on a Bernoulli experiment (in R: binom.test (k,n.p = 0.5,alternative = "two-sided ")).

## Supplementary Information

**Additional file 1.** 

**Additional file 2.** 

## Data Availability

The datasets supporting the conclusions of this article are included within the article and its additional files (Additional file 1.xlsx; Additional file 2.xls).

## Abbreviations

PGS(s): Polygenic score(s)
PGS_eGFR_crea_: Polygenic score based on 634 variants associated with eGFR_crea_
PGS_eGFR_cys_: Polygenic score based on 204 variants associated with eGFR_cys_
GWAS: Genome-wide association study
CAD: Coronary artery disease
CKD: Chronic kidney disease
eGFR_crea_/ eGFR_cys_: Glomerular filtration rate estimated by creatinine/ cystatin C
BMI: Body-mass- index
SNP: Single-nucleotide polymorphism
P: *P*-value
AF: Allele frequency
APOE: Apolipoprotein E
CST3: Cystatin C encoding gene
AD: Alzheimer´s disease
cpid: Variant identification by chromosome and position
rsID: Reference SNP cluster ID
EA: Effect allele
OA: Other allele
EAF: Effect allele frequency
