# Supplementary material for: Polygenic scores for estimated glomerular filtration rate in a population of general adults and elderly – comparative results from the KORA and AugUR study
Source: BMC Genom Data. 2023 May 25;24:28. doi: 10.1186/s12863-023-01130-9 (PMC10210501; doi:10.1186/s12863-023-01130-9)
Supplement: Supplementary file 1 — Additional file 1. [file 12863_2023_1130_MOESM1_ESM.pdf]

Supplemental Material for

Polygenic scores for estimated glomerular filtration rate in a population of general adults and elderly – comparative results from the KORA and AugUR study

Janina M. Herold, Jana Nano, Mathias Gorski, Thomas W. Winkler, Kira J. Stanzick, Martina E. Zimmermann, Caroline Brandl, Annette Peters, Wolfgang Koenig, Ralph Burkhardt, André Gessner, Iris M. Heid, Christian Gieger, Klaus J. Stark

Overview

Supplementary Tables

Supplementary Table 1: Overview of polygenic scores derived from published GWAS

Supplementary Table 2: Comparison of eGFRcys lowering alleles between elderly and general adults

Supplementary Table 2A: Effects of non- genetic variables on eGFR in the general adults

Supplementary Table 2B: Effects of non- genetic variables on eGFR in the elderly

Supplementary Table 3: PGS analyses for eGFR in the elderly and general adults considering further covariables

Supplementary Table 4: Comparison of eGFRcrea lowering alleles between elderly and general adults.

Supplementary Table 5: Comparison of eGFRcys lowering alleles between elderly and general adults.

Supplementary Table 6: PGS analyses for eGFR in the elderly and general adults excluding *APOE* variant.

Supplementary Table 7: Mapping of genotyped variants in AugUR.

Supplementary Figures

Supplementary Figure 1: Weighted polygenic score (PGS) for eGFRcrea and eGFRcys in the elderly and the general adults.

Supplementary Figure 2: Sensitivity analyses for non-linearity in model 1

Supplementary Figure 3: Distribution of the number of eGFR-lowering alleles in the polygenic risk scores in the general adults and the elderly

Supplementary Figure 4: Differences in allele frequencies between general adults and elderly

Supplementary Figure 5: QQ plots of variant associations with age and study membership

Supplementary Figure 6: Workflow of PGS analyses

Supplementary Methods

DNA extraction and genotyping in AugUR

Sample QC

Quality control of genotyped variants

Genotype imputation

Selection of variants and effect estimates

Variant extraction

R^2 as parameter for explained variance

Supplementary Tables

Supplementary Table 1. Overview of polygenic scores derived from published GWAS. For each set of variants underlying analyses and adjustment of variants for PGS inclusion (e.g. proxy variants) are given. Details for the variants are given in Supplementary Tables 6-11.

Phenotype	Variant set	Publication	Number of SNPs in publication	Source for beta estimates	SNPs in PGS (AugUR/KORA)
$eGFR_{crea}$	$eGFR_{crea}$ decreasing alleles	PMID: 34272381 (1)	634 single signals	European- restricted conditioned meta-analysis n= 1,004,040	634/633
$eGFR_{cys}$	Lead variants with $eGFR_{cys}$ lowering effect	Unpublished	205	$eGFR_{cys}$ meta-analysis n= 460,826	204/204

Supplementary Table 2a. Effects of non- genetic variables on eGFR in the general adults. Linear regression models for $eGFR_{crea}$, $eGFR_{cys}$ in KORA (n=2,900). Each line represents a single univariate model with respective regression coefficient (B) with 95% confidence interval (CI) and p-values for each dependent variable. Adjusted R^2 is given in percent as parameter for explained variance of total variance of phenotype.

Biomarker<br>Co-variables	B [95% CI]	p-value	R^2 (%)	eGFR variance
$eGFR_{crea}$ [mL/min/1.73 m^2]^2				239.92
Age [years]	-0.72 [-0.75, -0.68]	1.44×10^{-262}	33.8	
Men	1.18 [0.056, 2.31]	0.04	0.1	
BMI [kg/m²]	-0.57 [-0.68, -0.45]	2.30×10^{-20}	2.9	
Hypertension	-7.55 [-8.73, -6.37]	2.16×10^{-35}	5.1	
Antihypertensives	-12.16 [-13.75, -10.58]	1.65×10^{-49}	7.2	
High-ceiling diuretics	-16.83 [-21.74, -11.91]	2.33×10^{-11}	1.5	
CAD	-10.7 [-14.43, -5.72]	$6.01x10^{-6}$	0.7	
Diabetes	-8.72 [-11.98, -5.45]	1.84×10^{-7}	0.9	
Antidiabetics	-9.32 [-12.87, -5.76]	2.99×10^{-7}	0.9	
Education [years]	0.59 [0.37, 0.80]	$7.36x10^{-8}$	1.0	
Smoking (ever)	2.53 [1.39, 3.67]	$1.42x10^{-5}$	0.6	
$eGFR_{cys}$ [mL/min/1.73 m^2]^2				325.90
Age [years]	-0.94 [-0.98, -0.901]	1.00×10^{-320}	42.8	
Men	1.43 [0.11, 2.75]	0.033	0.12	
BMI [kg/m²]	-1.28 [-1.41, -1.14]	1.08×10^{-74}	10.9	
Hypertension	-11.01 [-12.36, -9.65]	4.73×10^{-55}	8.1	
Antihypertensives	-19.33 [-21.12, -17.55]	2.48×10^{-98}	13.5	
High-ceiling diuretics	-25.77 [-31.45, -20.07]	1.40×10^{-18}	2.6	
CAD	-12.14 [-17.22, -7.06]	$2.94x10^{-6}$	0.7	
Diabetes	-15.74 [-19.53, -11.95]	5.60×10^{-16}	2.2	
Antidiabetics	-15.60 [-19.73, -11.47]	1.72×10^{-13}	1.8	
Education [years]	1.35 [1.11, 1.60]	$7.56x10^{-27}$	3.9	
Smoking (ever)	0.89 [-0.45, 2.23]	0.19	0	

Sex is coded as “0” for women and “1” for men.

Supplementary Table 2b. Effects of non- genetic variables on eGFR in the elderly. Linear regression models for $eGFR_{crea}$, $eGFR_{cys}$ in AugUR (n=2,272). Each line represents a single univariate model with respective regression coefficient (B) with 95% confidence interval (CI) and p-values for each dependent variable. Adjusted R^2 is given in percent as parameter for explained variance of total variance of phenotype.

Biomarker<br>Co-variables	B [95% CI]	p-value	R^2 (%)	Phenotype variance
$eGFR_{crea}$ [mL/min/1.73 m²]²				255.86
Age [years]	-1.01 [-1.14, -0.89]	9.57×10^{-55}	10.1	
Men	-0.11[-1.21,1.43]	0.87	0	
BMI [kg/m²]	-0.604 [-0.75, -0.46]	7.26×10^{-16}	2.8	
Hypertension	-5.51 [-6.97, -4.06]	1.78×10^{-13}	2.3	
Antihypertensives	-7.85-(-9.22, -6.49]	1.16×10^{-28}	5.2	
High-ceiling diuretics	-15.42 [-17.29, -13.55]	7.32×10^{-56}	10.3	
CAD	-8.74 [-10.52, -6.95]	$1.81x10^{-21}$	3.9	
Diabetes	-6.38 [-7.98, -4.78]	7.84×10^{-15}	2.6	
Antidiabetics	-7.26 [-9.02, -5.51]	8.16×10^{-16}	2.8	
Education [years]	0.39 [0.21, 0.59]	$4.76x10^{-5}$	0.7	
Smoking (ever)	0.18 [-1.14, 1.51]	0.79	0	
$eGFR_{cys}$ [mL/min/1.73 m²]²				282.16
Age [years]	-1.36 [-1.48, -1.23]	2.93×10^{-91}	16.5	
Men	0.91 [-0.47, 2.30]	0.201	0	
BMI [kg/m²]	-0.85 [-1.01, -0.69]	2.95×10^{-27}	5.01	
Hypertension	-5.98 [-7.51, -4.45]	2.87×10^{-14}	2.5	
Antihypertensives	-8.97 [-10.401, -7.54]	1.0109×10^{-33}	6.2	
High-ceiling diuretics	-19.01 [-20.93, -17.09]	2.95×10^{-27}	14.2	
CAD	-8.36 [-9.91, -6.81]	$1.46x10^{-25}$	4.7	
Diabetes	-6.61 [-8.29, -4.99]	1.81×10^{-14}	2.5	
Antidiabetics	-7.05 -8.89, -5.20]	1.053×10^{-13}	2.4	
Education [years]	0.40 [0.20, 0.60]	$9.02x10^{-5}$	0.6	
Smoking (ever)	-0.41 [-1.80, 0.99]	0.57	0	

Sex is coded as “0” for women and “1” for men.

Supplementary Table 3. PGS analyses for eGFR in the elderly and general adults considering further covariables. In AugUR (70-95 years, n=2,272) and KORA S4 (20-69 years, n=2,900), we derived the PGS-association with $eGFR_{crea}$ and $eGFR_{cys}$ via linear regression, $Y_i = \beta_0 + \beta_1 PGS_i + \varepsilon_i, i = 1, \ldots, n$ with $\varepsilon_i \sim N(0, \sigma^2)$ independent and identically distributed. Y_i denotes the residuals of individual i adjusted for age, sex, BMI, diabetes, hypertension, CAD and high-ceiling diuretics intake (model 4[a]) and additionally for education and ever smoking derived from current or previous smoking (model 4[b]). Model was further adjusted for 10 principal components. Shown are the residual eGFR variance, the regression coefficients (beta) per one unit increase in the PGS with 95% confidence interval (CI) and p-values, and the R^2 of the PGS. One unit in the PGS corresponds to one eGFR-lowering allele of average eGFR-effect.

Model name Covariables[a]	Residual variance [ml/min/1.73 m²]²	Beta (PGS) [ml/min/1.73 m²] [95% CI]	p-value (PGS)	R^2 (PGS) [%] [95% CI]
$eGFR_{crea}$ AugUR				
Model 4[a]	198.70	-0.23 [-0.27, -0.19]	5.04×10^{-29}	5.3 [4.47, 6.13]
Model 4[b]	198.90	-0.23 [-0.28, -0.19]	4.31×10^{-29}	5.4 [4.54, 6.26]
$eGFR_{crea}$ KORA S4				
Model 4[a]	156.37	-0.29 [-0.32, -0.26]	5.76×10^{-66}	9.6 [7.62, 11.58]
Model 4[b]	155.04	-0.29 8-0.32, -0.26]	1.82×10^{-66}	9.7 [7.69, 11.71]
$eGFR_{cys}$ AugUR				
Model 4[a]	191.64	-0.31 [-0.37, -0.24]	2.34×10^{-19}	3.5 [3.11, 3.89]
Model 4[b]	191.11	-0.30 [-0.37, -0.24]	5.73×10^{-19}	3.4 [3.03, 3.77]
$eGFR_{cys}$ KORA S4				
Model 4[a]	175.89	-0.34 [-0.40, -0.29]	1.14×10^{-32}	4.7 [4.10, 5.30]
Model 4[b]	175.56	-0.34 [-0.40, -0.29]	2.32×10^{-32}	4.7 [4.10, 5.30]

[a]Model 4 as previously shown (Table 2)

[b]Model 4 extended by education years and smoking (ever smoking versus never smoking) as covariables

Supplementary Table 4. Comparison of $eGFR_{crea}$ lowering alleles between elderly and general adults. Frequencies of 534* $eGFR_{crea}$ associated alleles were analyzed between AugUR (n=2,272) and KORA (n=2,900).For analyses of allele frequencies only variants with imputation quality > 0.8 were included.

cpid	rsID	EA	OA	EAF_{AugUR}	EAF_{KORA}	ΔEAF	p_{diff}	Nearest gene[a]
19:45411941	rs429358	T	C	0.889252	0.867212	-0.02204	0.000153162	APOE
5:39401384	rs35969577	T	G	0.414385	0.454989	0.040604	0.000168231	DAB2
2:226933	rs3791221	G	A	0.361259	0.331573	-0.029686	0.000334769	SH3YL1
5:68739202	rs1183278	C	T	0.479362	0.453933	-0.025429	0.000372873	MARVELD2
11:2150444	rs2585	C	T	0.701676	0.679118	-0.022558	0.001587308	IGF2
12:65965572	rs4257020	T	C	0.781978	0.75132	-0.030658	0.003157914	MSRB3-AS1
2:18676265	rs4567937	A	G	0.319575	0.350053	0.030478	0.004418388	LOC105373454
9:95886051	rs3793662	C	T	0.197998	0.186114	-0.011884	0.005363171	NINJ1
16:53191470	rs12597487	C	C	0.306089	0.293559	-0.01253	0.006641148	CHD9
1:115236057	rs17602729	G	A	0.852881	0.867608	0.014727	0.006716578	AMPD1
6:133924856	rs2636596	C	T	0.788108	0.797254	0.009146	0.007845721	TARID
2:40680149	rs2301343	T	G	0.756232	0.736404	-0.019828	0.008759501	SLC8A1
15:79033534	rs12899201	A	G	0.749694	0.730201	-0.019493	0.009395435	-
5:34504277	rs13157326	A	G	0.500204	0.478089	-0.022115	0.010142553	-
1:243474536	rs2783971	A	C	0.4953	0.506468	0.011168	0.011649104	SDCCAG8
16:28857645	rs7187776	A	G	0.58725	0.607709	0.020459	0.01243956	TUFM
20:8315317	rs6055748	A	G	0.741316	0.715549	-0.025767	0.012719842	PLCB1
12:57116249	rs3782232	G	A	0.9288925	0.9399419	0.0110494	0.012886437	NACA
13:51160744	rs3118919	G	A	0.806498	0.782867	-0.023631	0.012929814	LOC107984567
20:2808307	rs6084180	T	C	0.786065	0.786167	0.000102	0.013309942	-
6:130374461	rs7740107	T	A	0.264405	0.270855	0.00645	0.014561803	L3MBTL3
1:56925754	rs115778011	C	T	0.0300368	0.0228353	-0.0072015	0.017409279	LOC124904185
7:26579648	rs3757657	A	G	0.402738	0.42661	0.023872	0.019616535	KIAA0087
3:53101640	rs11714876	G	C	0.0991009	0.094773	-0.0043279	0.020132854	RFT1
5:39426307	rs13179493	C	T	0.290356	0.305966	0.01561	0.020696951	DAB2

Table continued

cpid	rsID	EA	OA	EAF_{AugUR}	EAF_{KORA}	ΔEAF	p_{diff}	
16:84776178	rs7199123	T	T	0.300163	0.319034	0.018871	0.022736499	USP10
11:118966780	rs2509851	C	A	0.387209	0.38226	-0.004949	0.025212485	DPAGT1
1:228532195	rs417237	G	T	0.391091	0.410771	0.01968	0.027032485	OBSCN
6:131871605	rs2608916	G	T	0.212096	0.19971	-0.012386	0.027587189	-
4:96108805	rs3796463	G	C	0.297507	0.275475	-0.022032	0.031203289	UNC5C
10:124149352	rs7088058	C	T	0.606661	0.600053	-0.006608	0.035044987	PLEKHA1
3:30750404	rs6780429	A	C	0.538619	0.55689	0.018271	0.035991331	-
5:39436079	rs10077761	G	A	0.767879	0.790787	0.022908	0.037527906	-
4:115498457	rs71606723	T	A	0.239068	0.238648	-0.00042	0.03890852	UGT8
15:63580155	rs11071738	T	C	0.521659	0.533791	0.012132	0.043276917	APH1B
22:24168544	rs2330624	G	C	0.080507	0.07273	-0.007777	0.044387671	SMARCB1
2:135727188	rs6716446	G	A	0.309563	0.313358	0.003795	0.045237885	MAP3K19

cpid= variant identification by chromosome and position, rsID= reference SNP cluster ID, EA= effect ($eGFR_{crea}$ lowering allele), OA= other allele, EAF= effect allele frequency, ΔEAF= difference in allele frequency (EAF_{KORA} - EAF_{AugUR}, p-value resulted from chi-squared test; **variant reached Bonferroni corrected significance level (adjusted p-value= 0.05/534= 9.36×10^{-5}. [a]Genes were mapped using the database of genetic variation provided by the National Center for Biotechnology Information (NCBI) (PMID: 11125122).

Supplementary Table 5. Comparison of $eGFR_{cys}$ lowering alleles between elderly and general adults. Frequencies of 186* $eGFR_{cys}$ associated SNPs were analyzed between AugUR (n=2,272) and KORA (n=2,900). For analyses of allele frequencies only variants with imputation quality > 0.8 were included.

cpid	rsID	EA	OA	EAF_{AugUR}	EAF_{KORA}	ΔEAF	P_{diff}	Nearest gene
19:45411941	rs429358**	T	C***	0.889252	0.867212	-0.02204	1.53E-04	APOE
2:243929	rs55753056	A	G	0.331309	0.361259	-0.02995	3.82E-04	SH3YL1
5:39385641	rs2542713	A	C	0.466209	0.424397	0.041812	3.86E-04	DAB2
2:18681597	rs17694001	G	A	0.350185	0.318553	0.031632	2.40E-03	NT5C1B-RDH14
16:53375964	rs11860535	C	T	0.287751	0.29812	-0.010369	2.63E-03	CHD9
12:84089045	rs11115974	A	G	0.553458	0.537801	0.015657	1.34E-02	TMTC2
4:128094309	rs9307594	G	A	0.490232	0.510012	-0.01978	2.27E-02	INTU
7:26566970	rs1990100	T	C	0.416711	0.391295	0.025416	2.79E-02	KIAA0087
15:57830151	rs117047297	C	T	0.9779567	0.9844708	-0.0065141	3.11E-02	CGNL1
16:21089489	rs9930127	G	A	0.540655	0.516347	0.024308	3.13E-02	DNAH3
9:96923144	rs10993074	T	C	0.312962	0.295464	0.017498	4.03E-02	MIRLET7A1
6:81379924	rs2444820	C	G	0.456177	0.447487	0.00869	4.33E-02	BCKDHB
1:172366806	rs9286854	G	A	0.585533	0.596036	-0.010503	4.38E-02	DNM3
9:14116484	rs498010	C	T	0.828537	0.814058	0.014479	4.70E-02	NFIB
6:116384598	rs2104032	C	G	0.433474	0.447895	-0.014421	4.76E-02	FRK
3:141717736	rs9877536	C	G	0.678722	0.692889	-0.014167	4.87E-02	TFDP2
21:35358762	rs2834322	A	G	0.166843	0.180425	-0.013582	4.89E-02	LINC00649

cpid= variant identification by chromosome and position, rsID= reference SNP cluster ID, EA= effect ($eGFR_{cys}$ lowering allele), OA= other allele, EAF= effect allele frequency, ΔEAF= difference in allele frequency (EAF_{KORA} - EAF_{AugUR}, p-value resulted from chi-squared test; **variant reached Bonferroni corrected significance level (adjusted p-value= 0.05/186= 2.69×10^{-4}; ***risk allele for Alzheimer´s disease

Supplementary Table 6: PGS analyses for eGFR in the elderly and general adults excluding *APOE* variant. We calculated PGS using variants with genome-wide significant association with $eGFR_{crea}$ and $eGFR_{cys}$ excluding the *APOE* variant (rs429358) and derived the PGS association in AugUR (n=2,272) and KORA S4 (n=2,900) via linear regression, $Y_i = \beta_0 + \beta_1 PGS_i + \varepsilon_i, i = 1, \dots, n$ with $\varepsilon_i \sim N(0, \sigma^2)$ independent and identically distributed. Y_i denotes the residuals of individual i adjusted for i) age and sex (model 1).

$eGFR_{cys}$ Model name Covariables[a]	Residual variance [ml/min/1.73 m²]²	Beta (PGS) [ml/min/1.73 m²] [95% CI]	p-value (PGS)	R^2 (PGS) [%] [95% CI]
$eGFR_{crea}$ AugUR				
Model 1[a]	228.93	-0.23 [-0.28, -0.19]	4.80×10^{-25}	4.6 [3.95, 5.25]
Model 1[b]	228.93	-0.23 [-0.28, -0.19]	2.88×10^{-25}	4.6 [3.95, 5.25]
$eGFR_{crea}$ KORA S4				
Model 1[a]	157.65	-0.29 [-0.33, -0.26]	5.44×10^{-66}	9.6 [7.62, 11.58]
Model 1[b]	157.65	-0.29 [-0.33, -0.26]	4.99×10^{-66}	9.6 [7.62, 11.58]
$eGFR_{cys}$ AugUR				
Model 1[a]	234.68	-0.35 [-0.42, -0.27]	2.70×10^{-20}	3.6 [3.19, 4.01]
Model 1[b]	234.68	-0.35 [-0.42, -0.27]	6.11×10^{-20}	3.6 [3.19, 4.01]
$eGFR_{cys}$ KORA S4				
Model 1[a]	186.38	-0.35 [-0.41, -0.29]	3.42×10^{-32}	4.7 [4.10, 5.30]
Model 1[b]	186.38	-0.35 [-0.41, -0.29]	1.48×10^{-32}	4.7 [4.10, 5.30]

[a]Model 1 as previously shown

[b]Model 1 with weighted PGS excluding the *APOE* variant

Supplementary Table 7: Mapping of genotyped variants in AugUR. Variant mapping for both Illumina array versions of Infinium Global Screening Array-24 (GSAMD Version 1 and Version 3) used for genotyping in AugUR. Unmapped variants get chromosome 0 and were excluded before merging AugUR1 and AugUR2. Beside autosomal variants located on chromosomes 1-22, x- and y-chromosomal variants as well as variants in pseudo-autosomal regions on the x- chromosome (PAR) and mitochondrial (MT) variants are listed continuing the chromosome code.

Region	Chr Code	AugUR1 (GSA-24v1-0_A1_ClusterFile)	AugUR2 (GSAMD-24v3-0-EA_20034606_A1)
unmapped	0	7,387	147
autosomal	1-22	672,590	692,536
X	23	17,903	29,857
Y	24	1,480	5,038
PAR	25	578	1,235
MT	26	140	1,246
SUM		**700,078**	**730,059**

Supplementary Figures

Supplementary Figure 1. Weighted polygenic score (PGS) for eGFRcrea and eGFRcys in the elderly and the general adults. Shown are the distributions of the PGS based on 634 variants for $eGFR_{crea}$ (A, C) and 204 variants for $eGFR_{cys}$ (B, D) in AugUR (n=2,272; A, B) and KORA S4 (n=2,900; C, D) (left y-axis: number of individuals). Each PGS unit reflects an increase of one eGFR lowering allele of average genetic effect (x-axis). Also shown are mean values of $eGFR_{crea}$ and $eGFR_{cys}$ (CKD EPI 2009 (2), 2012 (3), respectively) and standard errors right y-axis) for each PGS category.

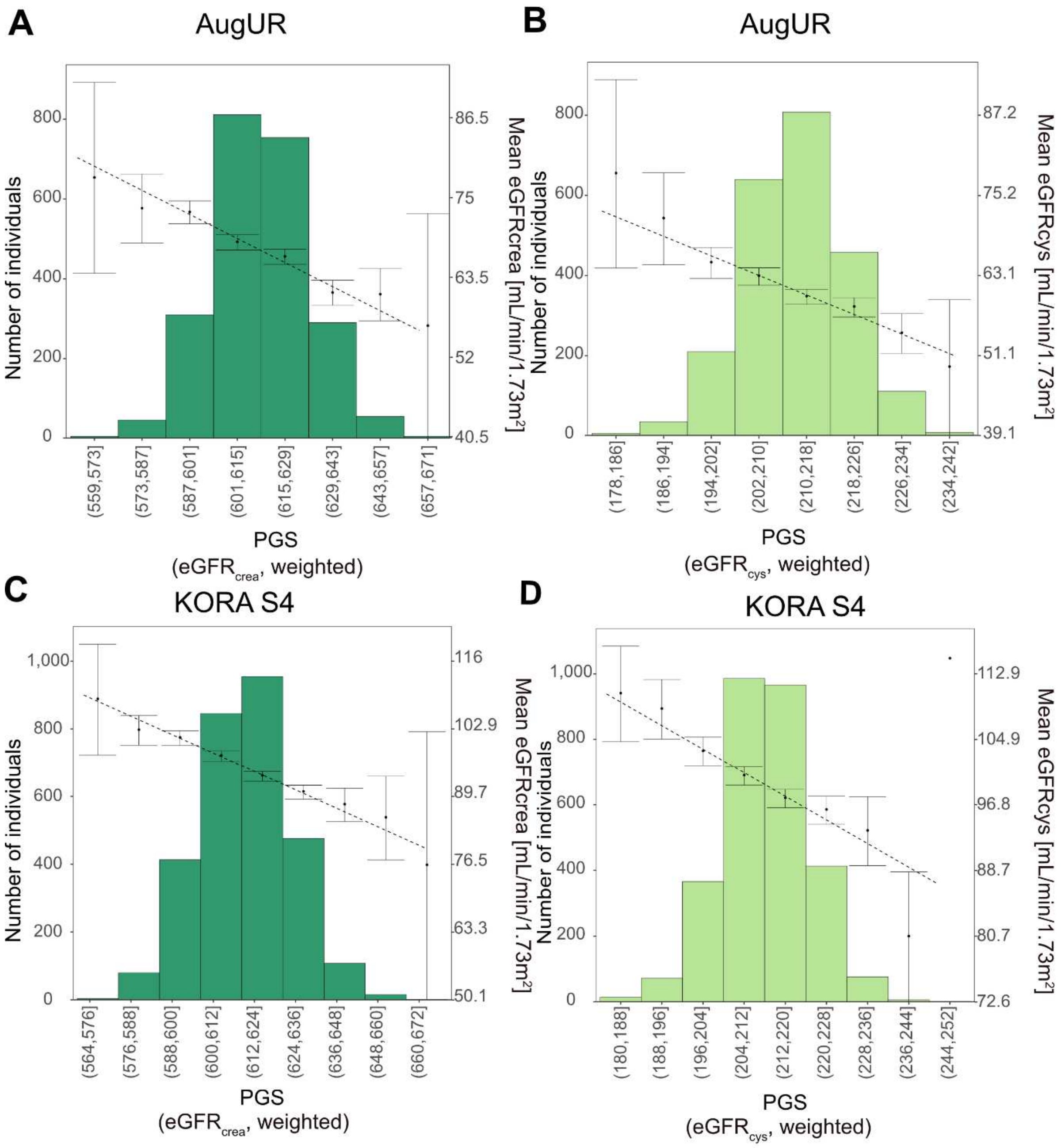

Supplementary Figure 2. Sensitivity analyses for non-linearity in model 1. Scatterplots show linear relation between residuals from linear regression models of eGFR residuals (derived from multiple linear regression on intercept, age, sex, and ten PCs) and weighted PGS (model 1). Shown are the residuals of $eGFR_{crea}$ (A, C) and $eGFR_{cys}$ (B, D) (y-axis) and predicted values (x-axis) in AugUR (n=2,272, A and B, respectively) and KORA S4 (n=2,900, C and D, respectively).

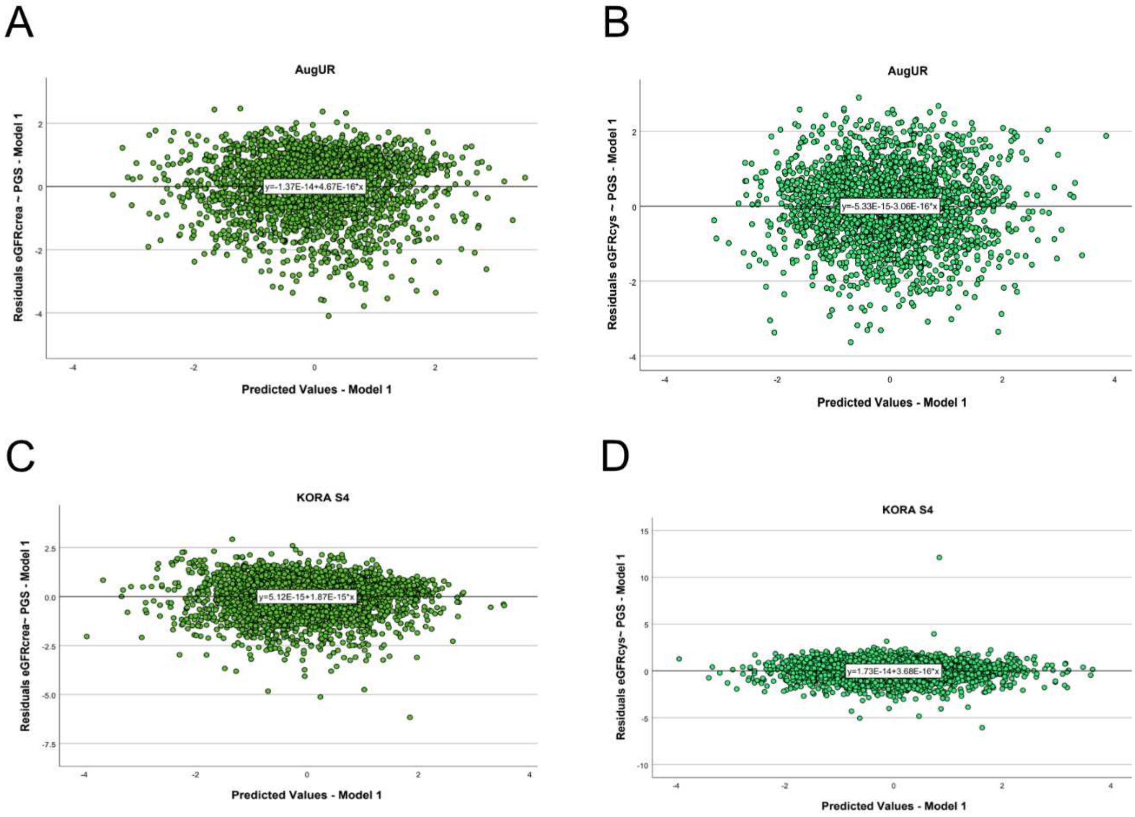

Supplementary Figure 3. Distribution of the number of eGFR-lowering alleles in the polygenic risk scores in the general adults and the elderly. In each of the general adults (KORA S4, n=2,900, magenta) and the elderly (AugUR, n=2,272, blue), we show the distribution of the number of the eGFR-lowering alleles among the 634 variant or 204 variant PGS for $eGFR_{crea}$ (A) or $eGFR_{cys}$ (B). Overlapping intervals are coloured in dark purple. The y-axis shows the relative frequencies of persons and the x-axis the number of $eGFR_{crea}$- or $eGFR_{cys}$-lowering alleles.

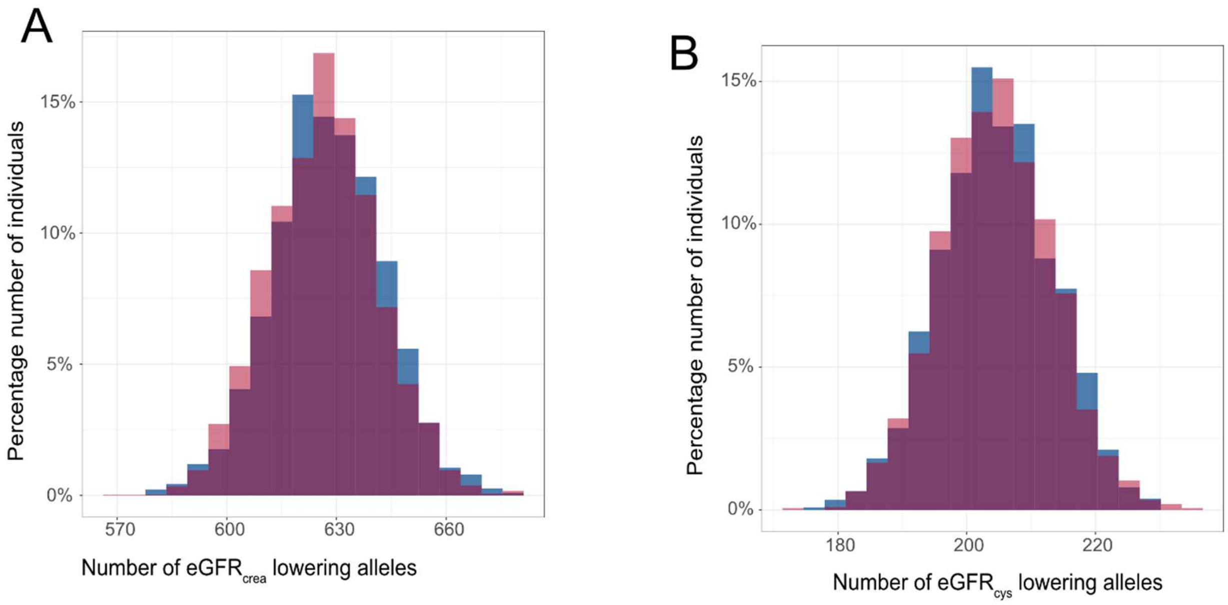

Supplementary Figure 4. Differences in allele frequencies between general adults and elderly. A. Frequencies of 534* $eGFR_{crea}$ (A) and 186* $eGFR_{cys}$ associated SNPs were analyzed between AugUR (n=2,272) and KORA (n=2,900) and expected p-values (x-axis) versus observed p-values (y-axis) were shown in a QQ-plot**. V**ariants are color coded according to p-values (p_{diff}) resulting from difference test (red: variant reached Bonferroni corrected significance level with adjusted p_{diff}= 0.05/534= 9.36×10^{-5} or 0.05/186= 2.69×10^{-4}, respectively; green: nominal significant p_{diff}; black: no significant p_{diff}). *For analyses of allele frequencies only variants with imputation quality > 0.8 were included.

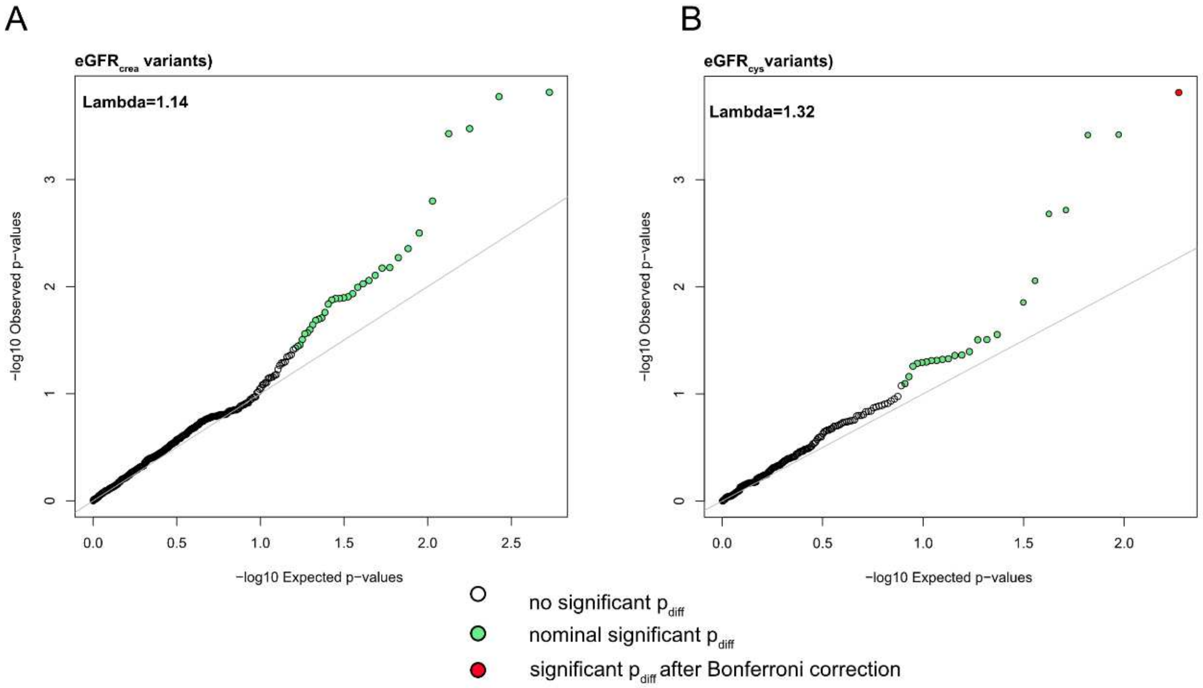

Supplementary Figure 5. QQ plots of variant associations with age and study membership. The data sets of AugUR (n=2,272) and KORA (n=2,900) were pooled and each of the 534 eGFRcrea-associated variants (imputation quality >0.8) were tested for association with age (unadjusted and adjusted for study membership) using linear regression and tested for association with study membership using logistic regression. Shown are expected versus observed association P-Values for the 534 eGFRcrea variants on study membership (A), age (B) and age adjusted for study membership (C) as well as for the 186 eGFRcys variants on study membership (D), age (E) and age adjusted for study membership (F). Details are given in Supplementary Tables 13 and 14.

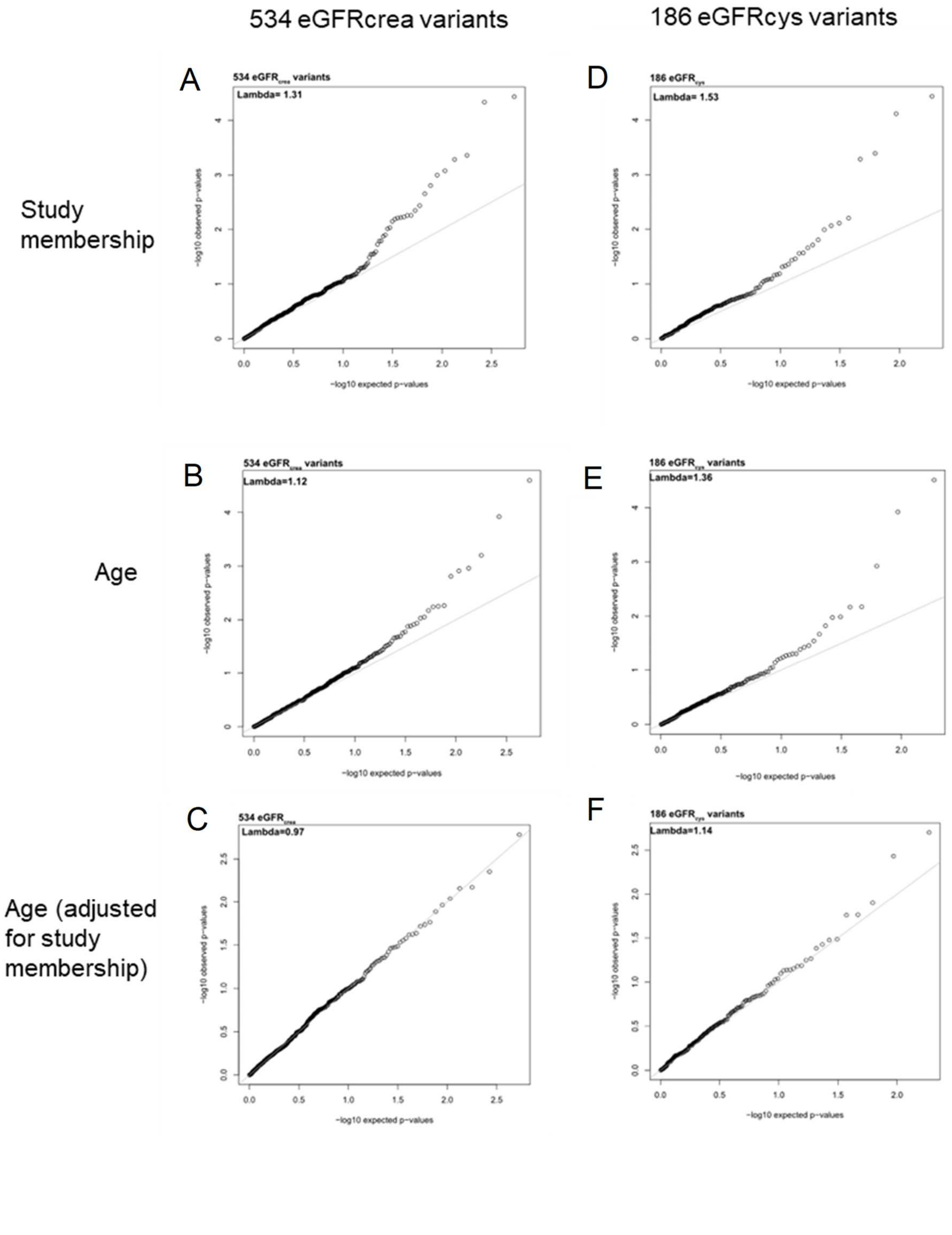

Supplementary Figure 6. Workflow of PGS analyses. Summary statistics from genome-wide association studies were used to derive eGFR- PGSs for HRC imputed genotypes in the AugUR study (n=2,272). Linear regression models were evaluated with focus on model quality and explained variance by PGS. Each point is explained in detail in the corresponding section in the text. PGS calculation and evaluation was simultaneously done for KORA S4 data.

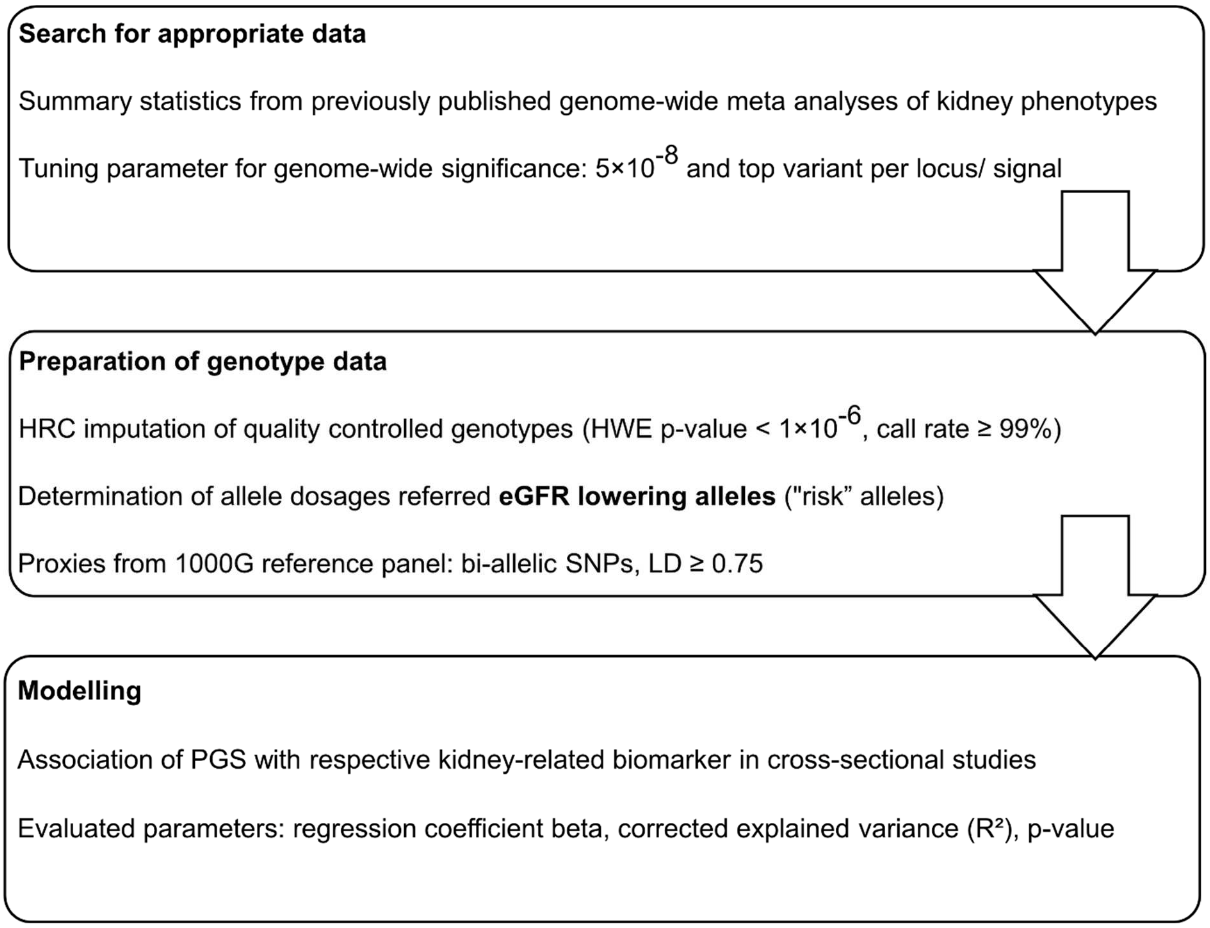

Abbreviations:
HRC=Haplotype reference consortium
HWE= Hardy-Weinberg equilibrium
LD reference panel=Linkage-disequilibrium based proxy tool of the National Cancer Institute

Supplementary Methods

DNA extraction and genotyping in AugUR. From whole blood ascertained at baseline visit and stored at -20°C, genomic DNA was extracted applying precipitation methods (Gentra Puregene Blood Kit, Qiagen, Hilden, Germany, and modified therefrom). After extraction, DNA was stored at -20°C in 10 mM Tris / 1 mM EDTA buffer, pH 8.0. For AugUR1, genotyping was conducted by the Genome Analysis Center, Helmholtz Zentrum Munich, Germany in September 2017 using the Infinium Global Screening Array-24, GSAMD, version 1.0 (Illumina Inc., San Diego, USA) with 700,078 variants before quality control. Variant calling was performed with the "GSAMD-24v1-0_A1_ClusterFile.egt" cluster file. For AugUR2, genotyping was conducted by Life & Brain GmbH, Bonn, Germany in October 2020 using the Infinium Global Screening Array-24, GSA, version 3.0 (Illumina Inc., San Diego, USA) with 730,059 variants before quality control (Supplementary Table 7). Variant calling was performed with the "GSAMD-24v3-0-EA_20034606_A1" manifest file. Illumina genotype data follow strand designation of "TOP" and "BOT" which is not directly comparable to "forward" and "reverse" or "+" and "-". To avoid strand inconsistencies between AugUR1 and AugUR2 genotyped on two different array versions of Illumina, strand information in both manifest files was converted to human genome reference forward strand (human reference genome chromosome build 37, 2009) using Perl (Version 5.34.0) and following *StrandScript* (https://github.com/seasky002002/Strandscript), which enables evaluation of Illumina genotyping array design and sequence based strand correction (4). Manifest files of each version were annotated by alignment of given sequence information and reference sequence before updating binary files containing genotypes of AugUR1 and AugUR2 by strand corrections if necessary. Thereby, the cutoff for the mismatch percentage of probe sequence and reference sequence was set at 0.2; variants with values below were flipped due to reference strand orientation. The array data include unmapped, autosomal, gonosomal and mitochondrial variants. Unmapped variants were removed running *StrandScript.* Subsets of both AugUR1 and AugUR2 were built for autosomal variants as well as for x-chromosomal variants stratified by sex. Quality control was performed for each data set using PLINK v.1.9 (5).

Sample QC. DNA sample quality was estimated by determining the heterozygosity rate in well-called, independent common autosomal markers in HWE per subject. All autosomal variants were linkage disequilibrium (LD)-pruned with a sliding window-algorithm method *indep* (PLINK v.1.9) with a window size of 50 variants, shifted by 5 variants and an LD threshold of $R^2 = 0.5$). The heterozygosity rate for each subject was calculated. All subjects exceeding or undergoing two standard deviations from the median heterozygosity across all subjects were recorded (5+36 subjects). No subject showed a call rate below 95%. The DNA samples were genotyped on 50 batches, each. No batch effect was measured. The genetic gender was estimate based on the homozygosity rates per subject on the X and Y chromosome and was supported by B-Allele-Frequency (BAF) and Log-R-Ratio (LRR) Plots of the X and Y chromosome. Eleven samples showed three copies of the X chromosome, and 14 samples showed partial loss of Y. Subjects with atypical sex chromosome configurations were recorded (6). Relatedness was calculated using KING (7) in the remaining 2,404 participants and third-degree relatives or closer (kinship coefficient $\varphi \geq 0.0884$) were marked in the data set (n=139). One known mother-daughter pair was confirmed. Three related clusters of three participants and one with five participants were identified and 61 other related pairs were detected as 1st and 2nd degree relatives. From each pair of individuals with kinship $\varphi \geq 0.0884$ (n=92), one index participant (with the highest genotype call rate) was kept for analysis (n=72 related participants excluded), yielding a data set with 2,332 participants. Ancestry was determined by merging the data with the Human Genome Diversity Project (HGDP) (8) reference samples and determining the principle components with the R-package *SNPRelate*. Cluster center coordinates for African Americans (AAM), South East Asian (SEA) and European (EUR) subjects were defined as the median PC1 and PC2 from all subjects defined as AAM, SEA or EUR in the HGDP data. General centroid coordinates were defined as the median of PC1 and PC2 of the former 3 cluster centers. A subject was classified as being AAM, SEA or EUR, if the distance of its PC1, PC2 was less than one third of the distance between one of the respective cluster centers (AAM, SEA or EUR) and the general centroid, else “other”. Four subjects were classified as

“other” and four as SEA after the definition explained above. All other subjects are classified as EUR. The participants with non-European ethnicity were marked and, to avoid population stratification, excluded from the present analyses on PGS (n=8). The final genotype data set included 2,324 unrelated, European individuals.

Quality control of genotyped variants. Starting with the quality control of 589,552 autosomal SNPs in AugUR1, 456 variants with a deviation from Hardy-Weinberg equilibrium ($p<1x10^{-6}$). 27,297 variants with a call rate below 95% and duplicated variants (n=106) were excluded stepwise. From 557,303 autosomal variants in AugUR2, 3,241 variants deviating from Hardy-Weinberg equilibrium ($p<1x10^{-6}$) or with a call rate below 95% (4,131 variants) as well as 2,415 duplicated variants were removed. 561,693 variants of AugUR1 passed quality control and were merged based on chromosome and position with 574,863 variants from AugUR2. The common data set comprising 621,512 autosomal variants and 2,447 individuals underwent quality control again. Monomorphic variants (MAC=1), variants with a deviation from Hardy-Weinberg equilibrium ($p<1x10^{-6}$) and variants with a call rate below 99% were excluded. Quality control for x-chromosomal variants was performed separately for both array data and repeated for the merged data. In AugUR1 1,260 variants and 333 in AugUR2 were excluded from *Xmales* because of a call rate below 95%; 1,077 variants in AugUR1 and 1,071 had a call rate below 95% or deviated from HWE with $p<1x10^{-6}$ and were removed before merging. 13,072 variants in *Xmales* are genotyped on both array versions and passed filtering for call rate (>95%). For *Xfemales* 12,737 variants remain for imputation after quality control. From the 658,535 genotyped variants (on autosomes, sex chromosomes and mitochondrial DNA) available on both versions of the GSA, 501,100 autosomal and 25,809 X-chromosomal variants remained for imputation. In addition, 717 variants on the Y chromosome, no PAR and 72 mitochondrial variants were kept in the data set after filtering for call rate (>95%) without imputation.

Genotype imputation. Genotype imputation. Genotypes that passed all QC filters, not duplicated, aligned by strand to the reference panel were imputed on basis of a two- step approach of imputation (9) with an imputation service (10). Variants that differ by more than

20% from the allele frequency in the reference panel are excluded. By this overall 501,099 variants were utilized for the imputation of the autosomes and 13,206 for the x- chromosome. The final genotype data comprised imputed genotypes coded as the estimated number of copies of a specified allele (allelic dosage) for 39,131,579 autosomal variants and 1,228,034 variants on x- chromosome.

Selection of variants and effect estimates. The genetic variants for the PGS are based on published GWAS for eGFR (1) with an extended number of associated variants and an expanded study size compared to previous data (Supplementary Table 8). AugUR study was not included in GWAS discovery sample set and appropriate for testing and validating PGS in an independent data set. Criteria for the variant selection were besides a genome-wide significant association with the phenotype (p-value ≤ 5×10-8) an independency of the lead variants given by r^2 of LD < 0.8.

Variant extraction. An assignment of the variants selected from GWAS data within the 2,272 imputed AugUR genotypes and 2,900 imputed genotypes of the KORA S4 study including European and unrelated subjects was done by merging the SNPs on basis of their *cpid*, a number with chromosome and base position combined. A step to check for missing variants, allele mismatches or duplicates comparing the two data sets was included. The absence of a corresponding variant in AugUR and KORA were replaced by proxy variants (Supplementary Tables 10b, 11b and 12b, 13b, respectively) with the highest R^2 as possible (≥0.75) which was found in the interactive online LDproxy Tool of the National Cancer Institute based on the 1000 Genome project (11). In case of allele mismatches potentially caused by strand flips, allele values were aligned with AugUR alleles. Variants with higher allele frequency in AugUR and better imputation quality were chosen among duplicated variants. Lists of the complete variant sets used for PGS calculation in AugUR and KORA are given in Supplementary Tables 10a ,11a and 12a, 13a, respectively. Extraction of selected variants from AugUR data was performed using *VCFtools 0.1.17* (12) filtering the relevant allele dosages for selected SNPs from imputed autosomal variants.

PGS calculation. In the SNP-based additive polygenic genetic model, let i denotes the number of risk alleles of trait associated SNPs j in one individual, and let β denote the weight of SNP j. The individual PGS is the sum of all effect alleles (unweighted PGS). A weighted PGS is computed by multiplying the dosage of each allele with the corresponding effect from GWAS summary statistics. Weighted PGS was scaled by dividing through the average of effect sizes of all variants included:

$$PGS\_unweighted = \sum_{j=i}^{n} dosage(i,j)$$

$$PGS\_weighted = \frac{\sum_{j=i}^{n} dosage(i,j)ß(j)}{\frac{1}{n}\sum_{j=i}^{n\,(\beta)} \beta}$$

i= SNPs associated with trait/disease, β= effect size of associated variant and n= number of SNPs, dosage (i, j) = number of copies of SNP i in the genotype of individual j.

Effect alleles in PGS are coded as eGFR- lowering alleles reflecting a higher risk for kidney function decline. Dosages of AugUR genotypes were adjusted on the effect alleles from GWAS results as reference and, if necessary, changed by subtracting the particular dosage from the value 2 as highest possible number of effect allele per variant to get the correct effect allele dosage for AugUR.

R^2 as parameter for explained variance. R^2 indicates the percentage of variance of dependent variables explained by independent variables and was deduced from squared Person´s correlation coefficient.

$$Explained\ variance\ (R) = \frac{\sum(x-\bar{x})\,(y-\bar{y})}{\sqrt{\sum(x-\bar{x})^2\ \sum(y-\bar{y})^2}}$$

To avoid inclusion of biased information, R^2 values are corrected considering the number of included individuals (n) in relation to number of added independent variables (p) in the model.

$$R_{corrected}{}^2 = 1 - (1 - R^2) * \frac{n-1}{n-p-1}$$

95% confidence interval for R-squared was derived from standard error as described in the equation below.

$$SE_{R^2} = \sqrt{\frac{4R^2(1-R^2)^2(n-p-1)^2}{(n^2-1)(n+3)}}$$

95% CI (R^2) = $R^2 \pm 2SE_R{}^2$

References

1. Stanzick KJ, Li Y, Schlosser P, Gorski M, Wuttke M, Thomas LF et al. Discovery and prioritization of variants and genes for kidney function in 1.2 million individuals. Nat Commun 2021; 12(1):4350.

2. Levey AS, Stevens LA, Schmid CH, Zhang YL, Castro AF, Feldman HI et al. A new equation to estimate glomerular filtration rate. Ann Intern Med 2009; 150(9):604–12.

3. Inker LA, Schmid CH, Tighiouart H, Eckfeldt JH, Feldman HI, Greene T et al. Estimating glomerular filtration rate from serum creatinine and cystatin C. N Engl J Med 2012; 367(1):20–9.

4. Wang J, Samuels DC, Shyr Y, Guo Y. StrandScript: evaluation of Illumina genotyping array design and strand correction. Bioinformatics 2017; 33(15):2399–401.

5. Purcell S, Neale B, Todd-Brown K, Thomas L, Ferreira MAR, Bender D et al. PLINK: a tool set for whole-genome association and population-based linkage analyses. Am J Hum Genet 2007; 81(3):559–75.

6. Turner S, Armstrong LL, Bradford Y, Carlson CS, Crawford DC, Crenshaw AT et al. Quality control procedures for genome-wide association studies. Curr Protoc Hum Genet 2011; Chapter 1:Unit1.19.

7. Manichaikul A, Mychaleckyj JC, Rich SS, Daly K, Sale M, Chen W-M. Robust relationship inference in genome-wide association studies. Bioinformatics 2010; 26(22):2867–73.

8. Cavalli-Sforza LL. The Human Genome Diversity Project: past, present and future. Nat Rev Genet 2005; 6(4):333–40.

9. Howie B, Fuchsberger C, Stephens M, Marchini J, Abecasis GR. Fast and accurate genotype imputation in genome-wide association studies through pre-phasing. Nat Genet 2012; 44(8):955–9.

10. Das S, Forer L, Schönherr S, Sidore C, Locke AE, Kwong A et al. Next-generation genotype imputation service and methods. Nat Genet 2016; 48(10):1284–7.

11. Machiela MJ, Chanock SJ. LDlink: a web-based application for exploring population-specific haplotype structure and linking correlated alleles of possible functional variants. Bioinformatics 2015; 31(21):3555–7.

12. Danecek P, Auton A, Abecasis G, Albers CA, Banks E, DePristo MA et al. The variant call format and VCFtools. Bioinformatics 2011; 27(15):2156–8.
